# Large-scale interspecific associations and ecological context shape communal roosts of Western jackdaw (*Coloeus monedula*)

**DOI:** 10.1371/journal.pone.0346626

**Published:** 2026-05-20

**Authors:** Iñigo Palacios-Martínez, Martina Carrete, Javier García, Hany Alonso, Juan Arizaga, Óscar Frías, Carlos Godinho, Dailos Hernández-Brito, Francisco Hortas, Jesús Martín-Zúñiga, Raymundo Montoya-Ayala, Jorge Mouriño, Antonio Román Muñoz, Juan M. Pérez-García, Javier Prieta, Javier Sanz, Laura Solé-Bujalance, Paulo Travassos, Diego Villanúa, José M. Zamora-Marin, Guillermo Blanco

**Affiliations:** 1 Department of Evolutionary Ecology, Museo Nacional de Ciencias Naturales, CSIC, Madrid, Spain; 2 Escuela de Doctorado UAM, Centro de Estudios de Posgrado, Universidad Autónoma de Madrid (UAM), Madrid, Spain; 3 Departament of Physical, Chemical and Natural Systems, Universidad Pablo de Olavide, Seville, Spain; 4 Grupo Ibérico de Anillamiento (GIA-León), León, Spain; 5 Sociedade Portuguesa para o Estudo das Aves (SPEA), Lisboa, Portugal; 6 Department of Ornithology, Aranzadi Sciences Society, Donostia, Spain; 7 Mediterranean Institute for Agriculture, Environment and Development, Laboratory of Ornithology, Global Change and Sustainability Institute, University of Évora, Évora, Portugal; 8 Department of Conservation Biology, Estación Biológica de Doñana, CSIC, Sevilla, Spain; 9 Department of Biology, Institute of Marine Research, Universidad de Cádiz, Cádiz, Spain; 10 Centro de Instrumentación Científica, Universidad de Granada, Granada, Spain; 11 Facultad de Estudios Superiores Iztacala UNAM. UBIPRO. Laboratorio de SIG y Análisis Espacial, Estado de México, México; 12 Sociedade Galega de Ornitoloxía, Santiago de Compostela, Spain; 13 Departamento de Biología Animal, Universidad de Málaga, Málaga, Spain; 14 Department of Applied Biology, Universidad Miguel Hernández, Elche, Spain; 15 Grupo Local SEO-Cáceres, Cáceres, Spain; 16 Sociedad Aragonesa de Gestión Agroambiental S.L.U. (SARGA), Zaragoza, Spain; 17 Institut Català d’Ornitologia, Museu de Ciències Naturals de Barcelona, Barcelona, Spain; 18 Laboratório de Ecologia Fluvial e Terrestre, Universidade de Trás-os-Montes e Alto Douro, Vila Real, Portugal; 19 Gestión Ambiental de Navarra (GAN-NIK), Pamplona, Spain; 20 Asociación de Naturalistas del Sureste (ANSE), Murcia, Spain; National Museums of Kenya, KENYA

## Abstract

Communal roosting in birds often involves complex interspecific interactions influenced by ecological, social, and environmental factors. We examined winter roosts (n = 232) of western jackdaws (*Coloeus monedula*) across the Iberian Peninsula to assess patterns of species composition, roost sharing, heterospecific abundance, and dominance. Most roosts (71.6%) were shared with other species, primarily corvids, starlings (*Sturnus* sp.), and cattle egret (*Ardea ibis*), with an average richness of 2.7 species per roost. Shared roosts hosted significantly more jackdaws than non-shared roosts, with anthropogenic factors (e.g., proximity to landfills and urban areas) positively influencing sharing, whereas elevation, temperature range, and forest cover had negative effects. Shared roosting with cattle egrets, wood pigeons, and starlings increased jackdaw abundance, whereas glossy ibises and high densities of starlings reduced it. Jackdaws were numerically dominant in over half of shared roosts, though dominance was context-dependent and influenced by the abundance of certain associates. These results reveal selective interspecific associations shaped by environmental context and flexible social roles, emphasizing the importance of conserving large communal roosts across diverse habitats to support overwintering bird communities, particularly declining species in open and agricultural landscapes.

## Introduction

Understanding how ecological and social factors shape group living remains a central question in behavioural ecology [1–6]. Social assemblages for overnight roosting or daytime resting occur across a broad range of taxa, from insects [7] to mammals [8,9], although this behaviour has been most extensively studied in birds [10,11]. Communal roosts in birds can range from small family units to aggregations of millions of unrelated individuals, depending on the species, season, and geographic region, and may be either mono- or multispecific [10,11]. Ecological constraints, demographic contexts, and social dynamics can influence the cost-benefit trade-offs underlying roost formation and size [11–13]. However, the ecological and social mechanisms shaping interspecific associations within communal roosts at broad spatial scales remain poorly understood.

The main hypotheses proposed to explain communal roosting in birds relate to the benefits of predator avoidance, energy conservation through reduced thermoregulation, increased foraging efficiency, facilitation of information exchange, social organization, and mating [4,10,11,14–16]. Mixed-species foraging flocks and communal roosts often consist of closely related species that share ecological requirements [17,18]. For instance, within corvids (Corvidae, Passeriformes) and parrots (Psittaciformes), several species commonly form mixed foraging flocks and roosts [10,14,19,20], suggesting that phylogenetic proximity influences ecological interactions and social cohesion as related species tend to exhibit similar survival strategies, facilitating coexistence and communication [5,11]. Nevertheless, these and other groups also form monospecific roosts, as well as mixed roosts with distantly related species that do not necessarily share foraging habitats or trophic resources [10,11,14].

The individual and collective decisions involved in the formation of mixed-species roosts, and the associated species composition, may be influenced by similar factors as those shaping monospecific roosts [11–13]. These decisions vary across space and time, depending on the local species pool and the relative abundances of potential associates. Accordingly, species composition and abundance within mixed roosts may differ for the same species across its geographic range, in response to environmental variation, seasonality, and landscape composition [17,21–23]. Such variability in social flexibility among species may shape roost-sharing patterns, which may also be modulated by ecological preferences, roost substrate availability and structure, predator presence, and daytime interspecific interactions in shared or exclusive foraging areas. Under conditions of food scarcity, unpredictability, or limited foraging time, such as winter in northern latitudes, species that are abundant, efficient at locating food, generalist in resource use, or proficient at avoiding predators may initiate associations that persist through the day and into nocturnal roosting [5,24–27]. Alternatively, mixed roosts may form among species that do not forage together but gain asymmetrical benefits from roosting proximity due to unequal predation risk or limited availability of optimal sites [28,29]. Roost-sharing may thus encompass a continuum from actively sought to incidental or forced interspecific associations [30,31].

Determining whether interspecific interactions among flock-mates are mutualistic, commensal, or instead impose costs on some species remains an ongoing challenge in behavioural ecology [1,32]. According to Mangini et al. [33], the nature of these associations may be shaped by the roles of species involved, namely: (1) nuclear species, contributing to group formation and/or cohesion without necessarily being the most abundant; (2) leaders, initiating movements within mixed-species flocks; (3) sentinels, providing alarm calls used by associates; and (4) followers, tracking the movements of others (i.e., leaders). Among these, leaders and followers are the most consistently identified roles, typically associated with differences in abundance [34–36], ecological traits (e.g., diet, body size, predation risk), and environmental conditions [34,37–40]. For example, insectivorous, small-bodied, actively foraging species are generally considered followers in mixed-species groups, in contrast to leaders, which are often larger, more abundant, or dominant species [17,18,41,42]. Variation in ecological plasticity among associated species can alter niche overlap, such that follower species may offset competition costs through the presence of leaders [43,44], though these associations does not necessarily confer reciprocal benefits for the leaders [33]. These interactions remain understudied in the context of communal roosts, and baseline information on species composition and frequency of associations at broad spatial scales is still scarce [45].

In this study, we examine interspecific associations within winter communal roosts of the western jackdaw (*Coloeus monedula*) across the Iberian Peninsula. By analysing patterns in roost composition and size we aim to identify the environmental, ecological, and social factors influencing the establishment of roosts used exclusively by jackdaws and those shared with other bird species. We hypothesized that ecological factors, such as the type of roosting substrates, habitat configurations providing foraging opportunities, and favourable climatic conditions, would promote roost-sharing behaviour in jackdaws, particularly with generalist heterospecifics. We also evaluate how species composition and the identity and abundance of co-roosting species in shared roosts affect the size of western jackdaw roosts, and how these effects are shaped by environmental factors. Specifically, our hypothesis predict that larger roost size of jackdaws will be favoured by higher abundances of co-roosting species, especially other ecologically versatile species. Understanding the contexts under which these associations emerge and vary can provide insights into the mechanisms that structure mixed-species social systems and the ecological processes underlying communal roosting dynamics.

## Materials and methods

### Fieldwork and sampling procedures

Data on the distribution and size of western jackdaw (hereafter jackdaw) roosts were obtained from a complete, simultaneous census conducted in Spain and Portugal. Prior to the simultaneous census, existing information from bibliographic sources and online platforms was compiled, and local experts were consulted. In the months leading up to the census, active roost locations were identified by tracking flocks at dusk from foraging areas, resting sites, or breeding colonies to pre-roosting aggregation sites (i.e., places where they aggregate before settling into the roost) using vantage points and car surveys. Initial trials were conducted to determine the best observation locations and approach routes after active roosts were identified. On 11–12 December 2021, a simultaneous census of every roost in the study area was carried out, with additional confirmatory counts between 9 and 13 December 2021. In order to estimate the size of each roost, individuals were counted during flight at dusk or dawn by recording birds entering or departing the roost location following standard census protocols [46]. Perched individuals were counted directly at roosts where visibility permitted, and counts were aided when needed by photographs and video recordings of flocks. Each count was carried out by one or more observers in constant communication to prevent duplicate records (see García and Blanco [47] for details).

The wintering population of jackdaws in Spain and Portugal (including the Spanish autonomous cities of Ceuta and Melilla in northern Africa) was estimated at a total of 109,163 individuals (range: 102,384–116,835) distributed across 238 roosts. Jackdaws that could not be assigned to any roost (daytime flocks) amounted to 1,439 individuals in 43 flocks, representing only 1.0% of the total number of birds counted. The mean roost size was 468 jackdaws, with a maximum of 6,250 individuals. One of the roosts was located within the area of influence of the city of Ceuta, in Smir (Morocco), and held 1,700 jackdaws. Of the roosts located, only those comprising ≥ 5 individuals were included in the analyses to avoid counting isolated pairs using breeding colonies as winter roosts, although this situation is exceptional on the Iberian Peninsula. In total, 232 roosts were considered on the Iberian Peninsula (continental Spain and Portugal), of which 166 were shared with other species, while 66 roosts were used exclusively by jackdaws. For each roost, UTM coordinates and the roosting substrate (tree, wetland vegetation, or other structures such as buildings, cliffs, or pylons) were recorded.

All other bird species observed at jackdaw roosts were identified and counted using the same protocol. In some cases, observers did not record total abundances; for these roosts (*n* = 21), only presence was noted. We distinguished species that shared the roost with jackdaws from those present nearby but not roosting at the same location. Species with crepuscular or nocturnal activity patterns (e.g., *Nycticorax nycticorax*), those present incidentally due to nesting in communal roosting sites of jackdaws (e.g., *Myiopsitta monachus*), or rare and occasional visitors not present throughout winter (e.g., *Platalea leucorodia*) were excluded from analyses. Due to survey timing and identification difficulties, both starling species present in the Iberian Peninsula (*Sturnus unicolor* and *S. vulgaris*) were pooled into a single category (*Sturnus* sp.).

### Ethics statement

Fieldwork and procedures were conducted under permits from the regional and national agency, and the owners of private properties, in accordance with the approved guidelines of the Ethics Committee of CSIC (CEBA-EBD-11–28).

### Explanatory variables

We considered environmental, topographic, and climatic descriptors potentially influencing whether jackdaw roosts were shared with other species. Explanatory variables included: (1) elevation, used as a general measure of environmental suitability since jackdaws and other species exploiting open lowlands and agricultural landscapes may be limited by high altitudes; (2) land use composition, including urban areas, crops, grassland, scrubland, woodland, and wetlands, which indicates foraging habitat suitability and the potential presence of roosting sites and substrates; (3) distance to the nearest landfill, serving as a proxy for food availability exploited by opportunistic and generalist species; (4) vegetation productivity, estimated using the Normalized Difference Vegetation Index (NDVI) as an indicator of productive foraging grounds [48]; and (5) bioclimatic variables, including temperature and precipitation, which act as environmental filters. All variables were assessed at two spatial scales, defined as circular areas around each roost with radii of 500 m (roost microhabitat) and 20 km (primary foraging area) (S1 Table).

Elevation data were obtained from the NASA Shuttle Radar Topography Mission (30 m resolution; SRTM 2013), while land use categories were extracted from CORINE Land Cover 2018 (CLC18; 100 m resolution), and grouped into broader land-cover classes, represented as cover percentage within each buffer of 500 m and 20 km (S1 Table). NDVI values were derived from MODIS/Terra Vegetation Indices (500 m resolution; [49]) for December 2021. Climatic variables, including mean precipitation and temperature, and temperature range (i.e., the difference between maximum and minimum mean values across grids within each buffer), were obtained from WorldClim v2.1 (1 km resolution; [50]). These multi-year averages were used to account for interannual variation while reflecting the consistent use of the same roosting sites by jackdaws across years [46]. Distance from each roost to nearest landfill were obtained from CLC18. To account for spatial autocorrelation between roosts, we calculated a spatial autocovariate (weighted average distance to neighbouring samples; [51]) using the autocov_dist function (*spdep* package v.1.3–3; [52]).

### Statistical analyses

To address the study objectives, we first assessed the environmental factors influencing whether jackdaw roosts were shared (n = 166) or non-shared (n = 66) with other species (Generalized Linear Model, GLM, binomial error distribution, logit link function) at the roost microhabitat (500m buffer) and primary foraging area (20 km buffer) scales. Second, we investigated whether the number of jackdaws per roost (roost size), was associated with environmental variables or affected by the presence (n = 166 roosts) and abundance (n = 145 roosts; in the remainder 21 roosts abundance of heterospecifics was not collected) of co-occurring species (GLMs, log-normal error distribution, log link function), restricting the analysis to species present in at least six roosts to ensure reliable inference. Third, we evaluated whether jackdaw roost size was associated with environmental variables or affected by the presence and abundance of heterospecifics (GLMs, log-normal error distribution, log link function) according to roosting substrate (tree, wetland vegetation or other), given that substrate use may differ among species depending on their ecological requirements. Fourth, to attempt isolating the effect of the presence and abundance of each associated species from that of other co-occurring taxa on jackdaw roost size, we also analysed separately the subset of roosts used by each heterospecific species (GLMs with the appropriate error distribution in each case). Finally, we explored the factors underlying jackdaw dominance (i.e., numerical predominance) in shared roosts (GLM, binomial error distribution, logit link function).

Prior to analyses, we computed a correlation matrix among explanatory variables (corrgram function of *corrgram* package v.1.14; [53]) to assess and avoid multicollinearity, based on the variance inflation factor (VIF) criterion (vif function of package *HH* v.3.1.51; [54]). Model selection was based on the Akaike Information Criterion corrected for small sample sizes (AICc) [55]. Within each set of models, models with ΔAICc (i.e., difference between model i and the top-ranked model) < 2 were considered alternatives and included in model averaging. However, since model averaging has been questioned [56,57], we also report the outputs of alternative models. An effect was considered to have no, weak, or strong support when the 95% confidence interval (CI) of its coefficients overlapped, barely overlapped, or did not overlap with zero, respectively. GLMs were fitted using the glmmTMB function of the *glmmTMB* package v.1.1.8 [58]. Model selection and averaging were performed using the model.sel and model.avg of the package *MuMIn* v.1.47.5 [59]. To assess model fit, we employed a simulation-based residual diagnostic approach (simulateResiduals function of *DHARMa* package v.0.4.6; [60]), generating standardized residuals and testing for dispersion, zero inflation, and overall goodness-of-fit (H₀: the fitted model adequately fits the data). The discriminatory power between shared and non-shared roosts was evaluated using the Kappa statistic of the best model at each spatial scale (confusionMatrix of the *caret* package v.6.0–94; [61]).

## Results

### Composition of shared roosts

**Fig 1:**
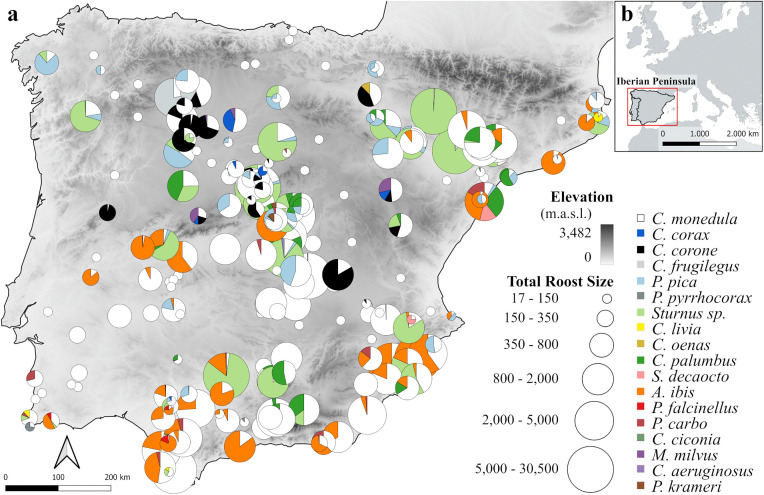
(a) Locations of western jackdaw (*Coloeus monedula*) roosts across the Iberian Peninsula. Pie charts represent the composition and relative proportions of co-roosting species (see legend; S1 Table provides detailed data). Non-shared roosts and roosts with unidentified heterospecifics are shown in white. Point size corresponds to total roost size. Background topography is based on a 30 m resolution digital elevation model from NASA Earth Observatory (http://earthobservatory.nasa.gov/). (b) Overview of the Iberian Peninsula (Spain and Portugal) indicating the general study area. Country borders were obtained from Natural Earth (https://www.naturalearthdata.com/).

A large proportion (71.6%, n = 232) of jackdaw roosts were shared with other species, with other corvids, starlings, cattle egret (*Ardea ibis*), and the wood pigeon (*Columba palumbus*) being the most frequently associated taxa depending on roost substrate (Table 1, S2 Table; Fig 1 and S1 Fig). Jackdaws were numerically dominant in most of these roosts, except in those shared with starlings and rooks (*Corvus frugilegus*) (S2 Table). The mean number of species per shared roosts was 2.7 ± 0.8 (S2 Table).

**Table 1:** Number of western jackdaw (*Coloeus monedula*) roosts and the percentage in which each associated bird species combination occurred, categorized by substrate type (trees, wetland vegetation, and other substrates such as buildings, cliffs, or pylons). Only species combinations observed three or more times in the full dataset are included, listed in descending order of frequency. Species codes and nomenclature follow the definitions provided in S1 Table.

Species combination	Trees	Wetlands	Others
*A. ibis*	8 (34.8%)	15 (65.2%)	
*P. pica*	17 (89.5%)	2 (10.5%)	
*P. pica* + *Sturnus* sp.	11 (78.6%)	3 (21.4%)	
*C. corone*	11 (91.7%)	1 (8.3%)	
*Sturnus* sp.	7 (87.5%)		1 (12.5%)
*C. palumbus* + *P. pica*	8 (100%)		
*A. ibis* + *P. carbo*	1 (16.7%)	5 (83.3%)	
*C. palumbus*	5 (83.3%)	1 (16.7%)	
*A. ibis* + *P. falcinellus*	4 (80%)	1 (20%)	
*A. ibis* + *Sturnus* sp.		5 (100%)	
*A. ibis + P. pica*	2 (50%)	2 (50%)	
*C. palumbus + P. pica + Sturnus* sp.	4 (100%)		
*C. palumbus + Sturnus* sp.	4 (100%)		
*P. carbo*	1 (25%)	2 (50%)	1 (25%)
*P. pyrrhocorax*			3 (100%)

### Factors influencing roost sharing

**Fig 2:**
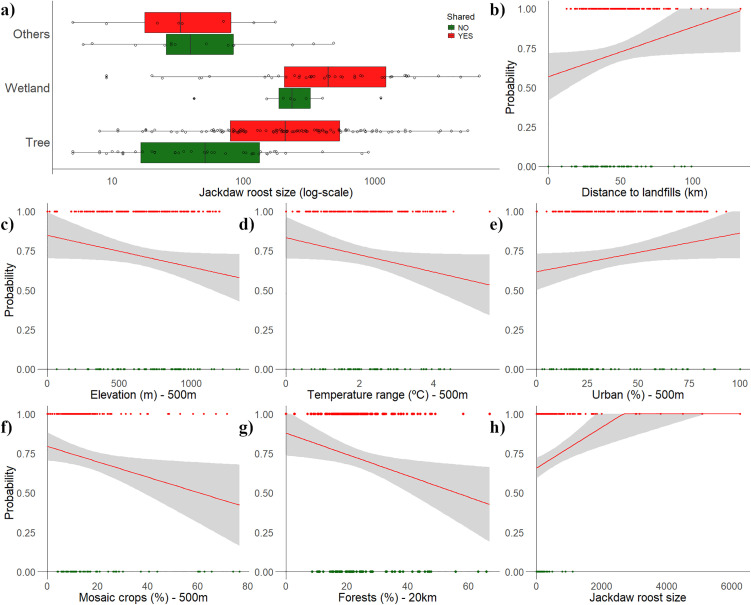
(a-h) Environmental drivers influencing the probability of roost sharing behaviour by western jackdaws (*Coloeus monedula*). (a) Mean (± SE) number of jackdaws at shared versus monospecific roosts located in trees, wetland vegetation, or other substrates. Green boxplots indicate monospecific roosts, and red boxplots indicate shared roosts. (b-h) Probability of jackdaw sharing a roost with other species with 95% confidence intervals, modelled as a function of jackdaw roost size, minimum distance to landfills, microhabitat characteristics (mean elevation, temperature range, proportion of urban areas, and mosaic crops within a 500 m buffer), and primary feeding areas (forest cover within a 20 km radius).

Most jackdaw roosts were located in trees (n = 158), followed by wetland vegetation (n = 52), and other substrates such as buildings, cliffs, or pylons (n = 22). Roost shared with other species were particularly frequent in trees (72.2% of roosts) and wetland vegetation (84.6%), whereas non-shared roosts predominated on other substrates (63.6%) (Table 2). Indeed, roost substrate was associated with whether roosts were shared or not (Table 2, S3 and S4 Tables; Fig 2a).

**Table 2:** Averaged GLM (binomial error) obtained to evaluate the effects of land-use, elevation, vegetation productivity and climatic variables measured in the immediate vicinity of roosts and within their primary foraging areas (500 m and 20 km buffers, respectively), as well as roost substrate type and distance to landfills on the probability that western jackdaws (*Coloeus monedula*) share winter roosts with other bird species in the Iberian Peninsula. Estimates and 95% confidence intervals (CI) were obtained by averaging all alternative models (ΔAICc < 2; see S3 Table). Significant effects (i.e., 95% CI not including zero) are bolded. This model explained 30.3% of the deviance. Variable names and details follow the definitions provided in S1 Table.

Variable	Estimate	2.5% CI	97.5% CI
**Intercept**	1.81	1.13	2.49
Roost substrate: Wetland	0.38	−0.69	1.45
**Roost substrate: Others**	−1.88	−3.02	−0.74
**Abundance of *C. monedula***	2.30	0.82	3.77
**Distance to landfills**	0.48	0.05	0.91
**Elevation 500m**	−0.51	−0.91	−0.12
**Temperature 500m**	−0.44	−0.82	−0.06
**Urban 500m**	0.44	0.03	0.84
**Mosaic crops 500m**	−0.39	−0.72	−0.06
Irrigated crops 20 km	0.21	−0.25	0.67
**Forests 20 km**	−0.46	−0.85	−0.07
Shrublands 20 km	−0.24	−0.61	0.13

Anthropogenic factors, including proximity to landfills and the extent of urban areas, increased the likelihood of roost sharing, whereas higher elevation and greater temperature ranges at the microhabitat scale (500 m) had significant negative effects (Tables 2, S3 and S4 Tables; Fig 2b-e). Additionally, mosaic cover in the immediate vicinity of roost sites and forested areas in primary foraging grounds also reduced the probability of sharing a roost (Table 2, S3 and S4 Tables; Fig 2f-g). The averaged model explained 30.3% of the total variance and showed strong predictive performance, correctly classifying 93.4% of shared roosts and 54.5% of non-shared roosts (κ = 0.52). All DHARMa diagnostic plots are shown in S2 Fig in Supplementary Material.

On average, shared roosts hosted four times more jackdaws (mean = 585 ± 959, range = 5–6,250) than exclusive roosts (mean = 137 ± 207, range = 5–1,108) (Table 2; Fig 2h), with considerable variation across substrates (Table 2, S3 and S4 Tables; Fig 2a). The highest numbers of jackdaws in shared roosts were recorded in wetland vegetation (mean = 889 ± 1,210) and trees (mean = 504 ± 851), while substantially fewer individuals were recorded in other substrates (mean = 58 ± 60).

### Influence of heterospecifics on jackdaw roost size

**Fig 3:**
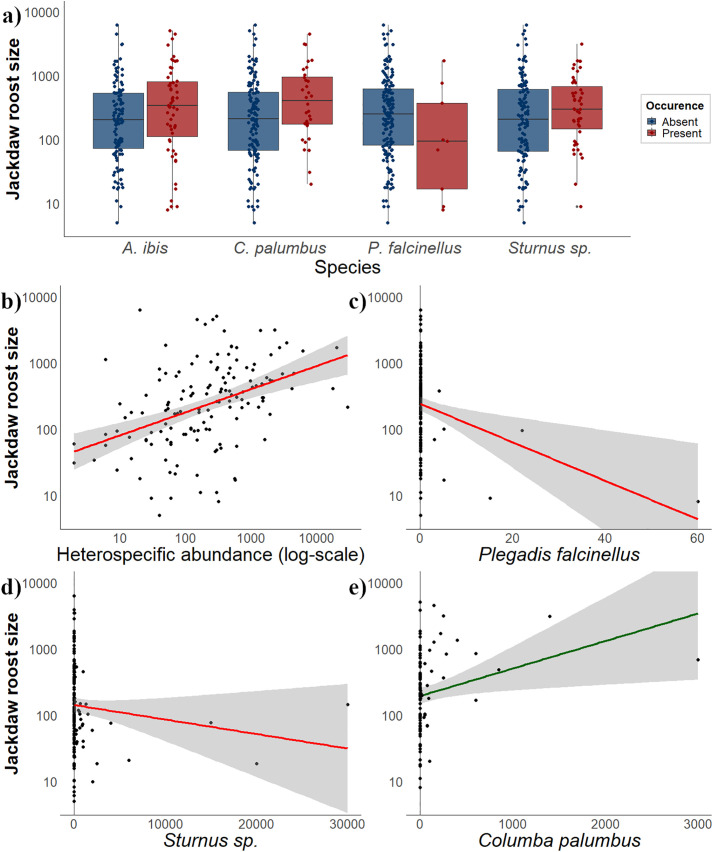
Responses of western jackdaw (*Coloeus monedula*) roost size at shared communal roosts (log-transformed) to significant heterospecific occurrences (a) and significant species-specific abundances (b–e). Panel (a) shows the mean (± SE) number of western jackdaws at shared roosts in the presence (red) or absence (blue) of western cattle egrets (*Ardea ibis*), wood pigeons (*Columba palumbus*), glossy ibises (*Plegadis falcinellus*), and starlings (*Sturnus* sp.). Panels (b–e) depict relationships between western jackdaw roost size and heterospecific abundances: (b) overall heterospecific abundance (log-scale), (c) glossy ibises, (d) starlings, and (e) wood pigeons only in tree groves. Regression lines represent correlations between jackdaw numbers and the explanatory variables, shown in red for all shared roosts and in green for roosts located in trees.

Jackdaw abundance in shared roosts was related to the presence of several avian species (Table 3; Fig 3a). Roosts where glossy ibises (*Plegadis falcinellus*) were present tend to host fewer jackdaws, whereas co-roosting with cattle egrets, wood pigeons, and starlings was associated with higher jackdaw numbers. Ravens (*Corvus corax*) showed a weak negative association (S5 and S6 Tables). The total abundance of heterospecifics within shared roosts was positively related to jackdaw counts (Table 3; Fig 3b). When examining individual species, high numbers of glossy ibises and starlings were associated with lower jackdaw abundances (Table 3; Fig 3c-d), while wood pigeons and cattle egrets showed weak positive relationships with jackdaw numbers (S7 and S8 Tables; Fig 3e-f). Averaged models based on heterospecific presence and abundance explained 15.3% and 13.2% of the total variance, respectively.

**Table 3:** Effects of the occurrence and specific abundances of co-roosting species on the size of western jackdaw (*Coloeus monedula*) winter roosts in the Iberian Peninsula (GLM, log-normal error distribution). For the occurrence model, co-roosting species were coded as presence/absence (1/0). For the specific abundance models, predictors included the total heterospecific abundance in each roost (i.e., all species other than jackdaws), the roost’s species richness, and the individual abundances of each species co-occurring with jackdaws. Estimates and 95% confidence intervals (CI) were obtained by averaging all alternative models with ΔAICc < 2 (see S7, S8 Tables). Significant effects (i.e., those with 95% CI not overlapping zero) are shown in bold. Variables that were significant in alternative models (S5, S6 Tables), but not in the averaged model, are marked with an asterisk (*). The averaged occurrence and specific abundance models explained 15.3% and 13.2% of the deviance, respectively. Variable names and details follow the definitions in S1 Tables.

Variable	Occurrence			Specific abundance		
	Estimate	2.5% CI	97.5% CI	Estimate	2.5% CI	97.5% CI
Intercept	4.95	4.45	5.45	5.37	5.13	5.62
Abundance of heterospecifics				1.44	**0.08**	**2.90**
Roost richness				0.09	−0.13	0.44
*P. falcinellus*	−1.43	**−2.45**	**−0.43**	−0.29	**−0.49**	**−0.10**
*A. ibis*	0.70	**0.13**	**1.27**	0.13	−0.25*	0.50
*Sturnus* sp.	0.51	**0.01**	**1.01**	−1.42	**−2.57**	**−0.27**
*P. pica*	−0.37	−0.97	0.03	0.07	−0.13	0.27
*C. palumbus*	0.76	**0.20**	**1.32**	0.17	−0.05*	0.40
*C. corone*	0.54	−0.17	1.24	−0.10	−0.29	0.10
*C. corax*	−0.73	−1.79	0.32*			
*P. carbo*	0.67	−0.09	1.42			
*M. milvus*	1.08	−0.31	2.52			

When analyses were conducted separately by roost substrate, the relationship between glossy ibis and jackdaw numbers was negative in both wetland vegetation and trees (S9 and S10 Tables). Wood pigeon abundance showed a consistent positive association across substrates (S9 and S10 Tables). In wetland vegetation, carrion crows (*Corvus corone*) were positively related to jackdaw numbers, while ravens showed a negative relationship (S10 Table). Across substrates, total heterospecific abundance showed a weak positive association with jackdaws only when roosting in trees (S11 Table), and starling numbers showed a weak negative association (S11 Table). Averaged models explained 14.1% of variance in trees and 39.7% in wetland vegetation. Finally, in species-specific subsets of shared roosts, the Eurasian magpie (*Pica pica*) was the only species showing a significant relationship with jackdaw numbers (S12 Table). All DHARMa diagnostic plots are shown in S2 Fig in Supplementary Material.

### Dominance in mixed-species roosts

**Fig 4:**
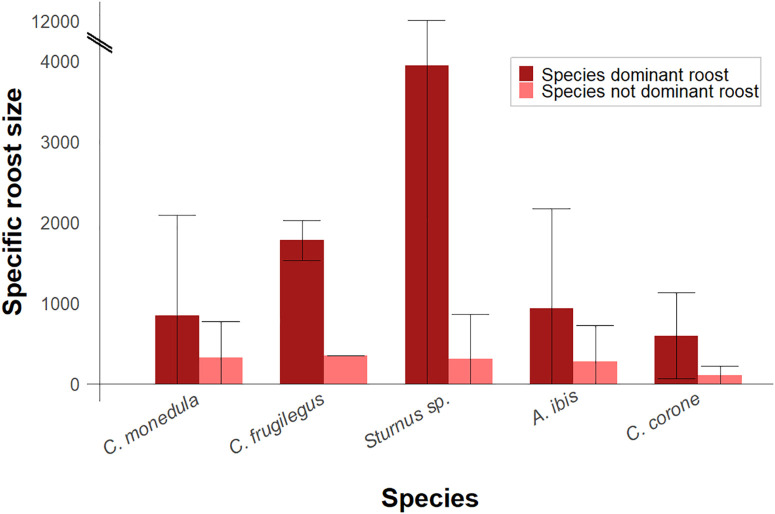
Mean relative abundances of the principal dominant co-roosting species in western jackdaw (*Coloeus monedula*) roosts under different numerical dominance conditions (see S13 Table). Bars represent the mean specific roost size ± SD when each species dominates (dark red) or does not dominate (light red) the communal roost shared by jackdaws, rooks (*Corvus frugilegus*), starlings (*Sturnus* sp.), cattle egrets (*Ardea ibis*), and carrion crows (*Corvus corone*). All species roost sizes under dominance and non-dominance conditions are provided in S13 Table.

Jackdaws were numerically dominant in more than half of the shared roosts (S13 Table), averaging 842 ± 1,244 individuals. When not dominant, jackdaw abundance dropped to 326 ± 442 individuals (S13 Table; Fig 4). Only starlings, rooks, and Eurasian collared doves (*Streptopelia decaocto*) were dominant in more than half of the roosts in which they occurred (S13 Table; Fig 4), whereas other commonly co-roosting species, such as great cormorants (*Phalacrocorax carbo*) and glossy ibises, were never dominant (S13 Table). Patterns were broadly similar across substrates: jackdaws were dominant in 55.8% of tree roosts, 52.8% of roosts in wetland vegetation, and 57.1% in other substrates.

Relationships between heterospecific abundances and dominance outcomes indicated that high numbers of starlings and cattle egrets were associated with a lower probability of jackdaw being the dominant species (S14 and S15 Tables). Carrion crow abundance showed a weak negative relationship with jackdaw dominance (S16 Table). Environmental predictors did not exhibit clear associations with jackdaw dominance status (S17and S18 Tables), although mean elevation in the immediate surrounding of roosts had a weak positive relationship (S19 Table). All DHARMa diagnostic plots are shown in S2 Fig in Supplementary Material.

Averaged models assessing jackdaw numerical dominance based on heterospecific abundance and environmental variables explained 32.7% and 5.8% of the total variance, respectively. The abundance-based model exhibited greater predictive capacity (κ = 0.54) than the environmental model (κ = 0.24), with both models performing better at predicting jackdaw dominance (correct classification: 91.3% and 78.8%, respectively) than roosts dominated by other species (60% and 55.4%, respectively).

## Discussion

Theoretical frameworks on the organization of mixed-species groups predict that species composition should emerge from the abundance of potential associates and from the social roles they adopt within aggregations [5,33,34]. Our study provides the first large-scale assessment of composition of winter communal roosts of jackdaws and shows patterns consistent with these expectations: interspecific associations are not random but instead aligned with ecological context and flexible social roles of the interacting species. Jackdaws frequently shared roosts with other corvids, starlings, cattle egrets, and wood pigeons supporting the prediction that species with overlapping ecological niches and compatible interspecific sociability can form mixed roosts [44,62,63]. Variation in species composition across roost substrates matched the ecological distribution of co-roosting species and the environmental features that influence their numerical dominance in both foraging areas and communal roosts. Because these features and species abundances vary geographically, mixed-roost composition is expected to shift across regions [23], as our large-scale dataset suggests. The frequent presence of widespread generalist species suggests that mixed roosts emerge where ecological compatibility and niche overlap permit the use of shared aggregation sites.

In addition to environmental correlates of roost sharing, our findings introduce a novel perspective: abundance-based dominance interacts with role-based dynamics to produce a context-dependent leadership within shared roosts. Larger jackdaw roosts were more frequently shared, suggesting that larger aggregations may act as interspecific attractors by enhancing dilution effects and information exchange [13,16,34,64]. In contrast, smaller monospecific roosts tended to occur in harsher environments, higher elevations and wider temperature ranges, where habitat suitability and heterospecific presence are limited in the Iberian Peninsula [65,66]. This pattern supports our hypothesis that mixed communal roosting is promoted by certain ecological characteristics shared by generalist species. Anthropogenic factors, particularly proximity to landfills and urban areas, were linked to higher roost sharing, reflecting the role of human-modified landscapes in providing favourable microclimates, reduced predation risk, and predictable food availability [67–70]. Nevertheless, because jackdaws predominantly forage in agricultural landscapes and rural matrices rather than urban cores [71–74] the scarcity of suitable substrates within urban areas may promote multispecies roosting [20,75,76]. Forest cover seems to limit shared roosting, supporting that open habitats that facilitate visual communication and information transfer may increase foraging efficiency among associated species [17,20,43]. Taken together, these results highlight that landscape configuration sets the structural bounds for multispecies aggregation.

Heterospecific occurrence and abundance significantly influenced jackdaw numbers. Roost sharing with cattle egrets, wood pigeons, and starlings was associated with larger jackdaw aggregations, supporting that widespread, abundant species that exploit similar foraging habitats are more likely to form large mixed roosts [17,18,71,72,77]. In contrast, smaller jackdaw numbers in roosts with glossy ibises may reflect competition for limited perching sites or disturbance from this much larger species at roost entrances late in the evening. Glossy ibises are locally abundant in wetlands for both roosting and foraging [78], forcing their overlap with jackdaws in regions where wetland vegetation is the only available roosting substrate. Corvids associates may benefit from cooperative vigilance or enhanced foraging opportunities among related species sharing ecological requirements [20,72,79,80]. Moreover, the ongoing winter urbanization of jackdaws [81] may also promote roost sharing with species already established in urban areas (e.g., the rose-ringed parakeet) and those that are expanding their winter presence in urban landscapes, including other corvid species [20].

The total abundance of heterospecifics had a significant positive effect on jackdaw roost size, supporting our hypothesis that larger jackdaw aggregations are favoured by higher abundances of co-roosters. In contrast, high abundances of glossy ibises reduced jackdaw numbers, further supporting a disruptive effect potentially mediated by disturbance under locally limited roosting conditions. Starlings, through their alarm calls, provided antipredator cues [82], that could influence jackdaw spatial behaviour and roosting patterns, potentially aiding predator detection if interpreted by jackdaws, as suggested by studies on interspecific use of alarm signals [83,84]. However, high concentrations of starlings could also attract predators, such as raptors specialized in capturing medium-sized prey [85,86], explaining the negative relationship between starling and jackdaw numbers. Occasional associations with less common species appeared neutral, suggesting mutual tolerance and substrate coincidence rather than strong ecological coupling. Overall, these patterns support that the costs and benefits of interspecific associations are highly context-dependent and shaped by species traits, abundances, and local environmental conditions. This context dependence also agrees with the flexible role framework, where species may shift between leader, follower, or nuclear roles depending on local conditions [5,33].

Jackdaws were numerically dominant in more than half of the mixed roosts and in all substrates, and this dominance varied systematically with the abundances of key associates. These patterns support that dominance within communal roosts should arise primarily from relative abundance rather than from fixed species traits. If dominant species disproportionately influence group cohesion or site selection, becoming leaders, then numerically subordinate species are expected to adopt follower roles and benefit from shared vigilance or improved information [34,35]. Our findings are consistent with this abundance-based framework: jackdaws, starlings, cattle egrets, and occasionally carrion crows appear able to alternate dominant roles according to their local numerical advantage. The broader implications of these patterns support the idea that species positions along a mutualism-antagonism continuum vary with ecological context, potentially producing conditional outcomes depending on local abundances and environmental constraints [1,43,44]. When jackdaws are dominant, they likely function as leaders or nuclear species, shaping roost cohesion and providing group-level cues relevant to foraging grounds and safe roosting sites. Conversely, when starlings or cattle egrets are numerically dominant, jackdaws may shift toward a follower role, integrating into pre-existing aggregations and potentially benefiting from their structure and daytime dynamics. These shifts highlight the flexibility of interspecific hierarchies within roosts and reflect abundance-dependent role allocation [26,33]. Understanding how these dynamics translate into fitness consequences for each species will require dedicated behavioural studies, as the net outcomes of these associations remain largely unresolved.

A central limitation of our study is that heterospecific relationships were evaluated using information restricted to jackdaw roosts. Therefore, we cannot identify species that jackdaws may avoid, or quantify associations at abundance levels or species combinations not represented in our sample. Thus, the effects of interspecific interactions might differ depending on the abundance of certain species beyond the range observed in shared roosts, on specific substrates, or in other combinations of associated species. While the presence or abundance of one species may influence another, the unknown locations and numbers of roosts not shared with jackdaws mean that observed relationships must be interpreted with caution. Additionally, the regional scope of our dataset limits generalization beyond the Iberian Peninsula, though they provide a valuable first approximation of large-scale social organization in jackdaw roosts. These constraints highlight the need for comprehensive, multi-species roost-network studies to test hypotheses about formation, structure and dynamics of heterospecific roosts.

In conclusion, jackdaw communal roosts represent dynamic interspecific networks shaped by local abundances, and environmental conditions. Our findings support hypotheses predicting abundance-based dominance, context-dependent social roles, and flexible interspecific interactions as major factors underlying structural patterns in mixed-species roosting. Through numerical strength, social tolerance and substrate versatility, jackdaws appear to influence roost structure in ways that likely benefit co-roosting species through increased safety and information sharing. However, the dramatic and rapid decline of jackdaw populations across the Iberian Peninsula [81,87] raises concerns about the stability of these multispecies networks. Protecting key roosting habitats, rural groves, wetlands, and peri-urban woodlands, may therefore be essential not only for jackdaws but also for other declining species of open and agricultural landscapes. Future research integrating behavioural interactions, vigilance dynamics, and temporal roost changes will be crucial for testing mechanistic hypotheses underlying these complex social systems. More broadly, integrating numerical dominance and flexible social role theory into studies of communal roosting offers a powerful framework for understanding how mixed-species groups form, persist, and respond to environmental change.

## Supporting information

S1 Table Variables used to assess factors influencing the presence and size of western jackdaw (*Coloeus monedula*) roosts in the Iberian Peninsula. Definitions of land uses are available online in the CORINE Land Cover guidelines (https://land.copernicus.eu/content/corine-land-cover-nomenclature-guidelines/docs/pdf/CLC2018_Nomenclature_illustrated_guide_20190510.pdf).
(PDF)

S2 Table Species recorded sharing winter communal roosts with the jackdaw (*Coloeus monedula*). This table presents all bird species observed co-occurring with jackdaws in winter communal roosts. For each species, the table includes: the scientific names; the percentage of shared roosts in which the species was present, and with the percentage of occupied roosts by substrate type (Tree / Wetland / Other); the mean specific abundance ± standard deviation (SD) within the roosts they occupied each species, and the mean ± SD of the species’ prevalence within jackdaw roosts (i.e., its percentage contribution to total roost size); the mean ± SD species richness in jackdaw roosts where the species occurred; the mean ± SD number of jackdaws present in those roosts, and the mean ± SD of the jackdaw’s proportional contribution to roost size. Species marked with an asterisk (*) are those that regularly formed roosts and were included in the analyses. Species detected in only six or fewer roosts were excluded from analyses. Variable names and details follow the definitions in S1 Table.
(PDF)

S3 Table GLM (binomial error) model selection of western jackdaw (*Coloeus monedula*) roost sharing at both scales (500 m and 20 km) (ΔAICc < 2). The null model was included in our set of models. df: degrees of freedom; AICc: Akaike information criterion corrected for small sample sizes; ΔAICc: difference between the AICc of model i and that of the best model (i.e., the model with the lowest AICc); w: Akaike weight.
(PDF)

S4 Table Alternative binomial GLM models explaining western jackdaw (*Coloeus monedula*) roost sharing in relation to environmental variables measured at two spatial scales (500 m and 20 km), with model support defined by ΔAIC < 2. Estimates and 95% confidence intervals were assessed. In bold, effects that received significant support (i.e., the 95% CI does not overlap zero).
(PDF)

S5 Table GLM (log-normal error) model selection of western jackdaw (*Coloeus monedula*) roost size in relation to the presence/absence of co-roosting species in the Iberian Peninsula (ΔAICc < 2). The null model was included in our set of models. df: degrees of freedom; ΔAICc: difference between the AICc (Akaike information criterion corrected for small sample sizes) of model i and that of the best model (i.e., the model with the lowest AICc); w: Akaike weight.
(PDF)

S6 Table Alternative log-normal GLM models explaining western jackdaw (*Coloeus monedula*) roost size in relation to the presence/absence of co-roosting species in the Iberian Peninsula, with model support defined by ΔAIC < 2. Estimates and 95% confidence intervals are shown. In bold, effects that received significant support (i.e., the 95% CI does not overlap zero).
(PDF)

S7 Table GLM (log-normal error) model selection of western jackdaw (*Coloeus monedula*) roost size in relation to the specific abundances of co-roosting species in the Iberian Peninsula (ΔAICc < 2). The null model was included in our set of models. df: degrees of freedom; ΔAICc: difference between the AICc (Akaike information criterion corrected for small sample sizes) of model i and that of the best model (i.e., the model with the lowest AICc); w: Akaike weight.
(PDF)

S8 Table Alternative log-normal GLM models explaining western jackdaw (*Coloeus monedula*) roost size in relation to the specific abundances of co-roosting species in the Iberian Peninsula, with model support defined by ΔAIC < 2. Estimates and 95% confidence intervals are shown. In bold, effects that received significant support (i.e., the 95% CI does not overlap zero).
(PDF)

S9 Table GLM (log-normal error) model selection of western jackdaw (*Coloeus monedula*) roost size in relation to the specific abundances of co-roosting species in the Iberian Peninsula, subdivided into the different substrates (ΔAICc < 2). The null model was included in our set of models. df: degrees of freedom; AICc: Akaike information criterion corrected for small sample sizes; ΔAICc: difference between the AICc of model i and that of the best model (i.e., the model with the lowest AICc); w: Akaike weight.
(PDF)

S10 Table Model averaging of all alternative log-normal GLM models (ΔAICc < 2) of roosting jackdaw (*Coloeus monedula*) roost size in relation to the specific abundances of co-roosting species in the Iberian Peninsula by roost substrate types. Estimates and 95% confidence intervals were assessed. In bold, effects that received significant support (i.e., the 95% CI does not overlap zero). (*) indicates the variables that were significant in some alternative models but not in the average model. Variances explained: Tree = 14.06%; Wetland = 39.70%; Other = 0.0%.
(PDF)

S11 Table Alternative log-normal GLM models explaining western jackdaw (*Coloeus monedula*) roost size in relation to the specific abundances of co-roosting species in the Iberian Peninsula, subdivided into the different substrates, with model support defined by ΔAIC < 2. Estimates and 95% confidence intervals were assessed. In bold, effects that received significant support (i.e., the 95% CI does not overlap zero).
(PDF)

S12 Table GLM models of univariant jackdaw roost size by specific abundances of co-roosting species (ΔAIC < 2). Estimates and 95% confidence intervals were assessed. In bold, effects that received significant support (i.e., the 95% CI does not overlap zero). Additionally, model error distributions and the proportion of variance explained (R²) were evaluated for each model.
(PDF)

S13 Table Summary of dominant species across communal roosts, including the percentage of roosts in which each species was dominant when each species is present, average jackdaw abundance, and average species-specific roost size when each species dominates or not the shared communal roost. (PDF)

S14 Table GLM (binomial error) model selection of roosting dominance by western jackdaws (*Coloeus monedula*) (1) or other species (0) in relation to specific abundances of co-roosting species in the Iberian Peninsula (ΔAICc < 2). The null model was included in our set of models. df: degrees of freedom; AICc: Akaike information criterion corrected for small sample sizes; ΔAICc: difference between the AICc of model i and that of the best model (i.e., the model with the lowest AICc); w: Akaike weight.
(PDF)

S15 Table Model averaging of all alternative binomial GLM models (ΔAICc < 2) of roosting dominance by western jackdaws (*Coloeus monedula*) (1) or other species (0) in relation to specific abundances of co-roosting species in the Iberian Peninsula. Estimates and 95% confidence intervals were assessed. In bold, effects that received significant support (i.e., the 95% CI does not overlap zero). (*) indicates the variables that were significant in some alternative models but not in the average model. The deviance explained by the averaged model is 32.72%.
(PDF)

S16 Table Alternative binomial GLM models explaining roosting dominance by western jackdaws (*Coloeus monedula*) (1) or other species (0) in relation to specific abundances of co-roosting species in the Iberian Peninsula, with model support defined by ΔAIC < 2. Estimates and 95% confidence intervals were assessed. In bold, effects that received significant support (i.e., the 95% CI does not overlap zero).
(PDF)

S17 Table GLM (binomial error) model selection of roosting dominance by western jackdaws (*Coloeus monedula*) (1) or other species (0) in relation to environmental features at different scales (500 m and 20 km) in the Iberian Peninsula (ΔAICc < 2). The null model was included in our set of models. df: degrees of freedom; AICc: Akaike information criterion corrected for small sample sizes; ΔAICc: difference between the AICc of model i and that of the best model (i.e., the model with the lowest AICc); w: Akaike weight.
(PDF)

S18 Table Model averaging of all alternative binomial GLM models (ΔAICc < 2) of roosting dominance by western jackdaws (*Coloeus monedula*) (1) or other species (0) in relation to environmental features at different scales (500 m and 20 km) in the Iberian Peninsula. Estimates and 95% confidence intervals were assessed. In bold, effects that received significant support (i.e., the 95% CI does not overlap zero). (*) indicates the variables that were significant in some alternative models but not in the average model. The deviance explained by the averaged model is 5.83%.
(PDF)

S19 Table Alternative binomial GLM models (ΔAICc < 2) of roosting dominance by western jackdaws (*Coloeus monedula*) (1) or other species (0) in relation to environmental features at different scales (500m and 20 km) in the Iberian Peninsula. Estimates and 95% confidence intervals were assessed. In bold, effects that received significant support (i.e., the 95% CI does not overlap zero).
(PDF)

S1 Fig Map of jackdaw shared roosts of the associated species. Point size is proportional of the overall roost size of the abundance of all roost abundance, and the colour corresponded to the different species within the jackdaw roosts. Country borders were obtained from Natural Earth (https://www.naturalearthdata.com/).
(TIFF)

S2 Fig DHARMa residual diagnostics for each analysis set's first alternative model: (a) binomial GLM evaluating jackdaw roost sharing at two spatial scales (500 m and 20 km) in relation to environmental variables; (b) log-normal GLM for jackdaw roost size in relation to co-roosting species abundances; (c) log-normal GLM for roost size in relation to co-roosting species presence/absence; (d) log-normal GLM for roost size in tree substrates as a function of co-roosting species abundances; (e) log-normal GLM for roost size in wetland substrates based on co-roosting species abundances; (f) binomial GLM for roosting dominance in relation to co-roosting species abundances; and (g) binomial GLM for roosting dominance in relation to environmental variables at two spatial scales (500 m and 20 km). (TIFF)
