# Supplementary material for: Large-scale interspecific associations and ecological context shape communal roosts of Western jackdaw (Coloeus monedula)
Source: PLoS One. 2026 May 20;21(5):e0346626. doi: 10.1371/journal.pone.0346626 (PMC13189308; doi:10.1371/journal.pone.0346626)
Supplement: S1 Table — Definitions of land uses are available online in the CORINE Land Cover guidelines (https://land.copernicus.eu/content/corine-land-cover-nomenclature-guidelines/docs/pdf/CLC2018_Nomenclature_illustrated_guide_20190510.pdf). (PDF) [file pone.0346626.s001.pdf]

S1 Table. Variables used to assess factors influencing the presence and size of western jackdaw (*Coloeus monedula*) roosts in the Iberian Peninsula. Definitions of land uses are available online in the CORINE Land Cover guidelines (https://land.copernicus.eu/content/corine-land-cover-nomenclature-guidelines/docs/pdf/CLC2018_Nomenclature_illustrated_guide_20190510.pdf).

**Variable**	
Urban areas	Continuous urban fabric
	Discontinuous urban fabric
	Industrial or commercial units
	Road and rail networks and associated land
	Port areas
	Airports
	Mineral extraction sites
	Construction sites
	Green urban areas
	Sport and leisure facilities
	Green urban areas
Dry crops	Non irrigated arable land
	Annual crops associated with permanent crops
Irrigated crops	Permanently irrigated land
	Rice fields
Woody crops	Vineyards
	Fruit trees and berry plantations
	Olive groves
Mosaics crops	Complex cultivation patterns
	Land principally occupied by agriculture with significant areas of natural vegetation
	Agro-forestry areas
Grasslands	Pastures
	Natural grasslands
Forests	Broad leaved forests
	Coniferous forests
	Mixed forests

Shrublands	Sclerophyllous vegetation<br>Transitional woodland shrub<br>Moors and heathland
Wetlands	Peat bogs<br>Inland marshes<br>Wetlands<br>Water bodies
Temperature	Temperature range December (2015-2020) (ºC)
Precipitation	Mean precipitation December (2015-2020) (mm)
NDVI	Landsat Normalized Difference Vegetation Index
Elevation	Mean elevation (m)
Substrate	Tree<br>Wetland (Reedbed, marshlands)<br>Other (buildings, cliffs, others)
Distance to landfills	Minimum distance to nearest dump (m)
AC	Spatial autocorrelation
Het_abundance	Heterospecific abundance in jackdaw roost
C. monedula	Western jackdaw (*Coloeus monedula*)
Richness	Roost richness (not counting jackdaws)
C. corone	Carrion crow (*Corvus corone*)
C. corax	Northern raven (*Corvus corax*)
C. frugilegus	Rook (*Corvus frugilegus*)
P. pyrrhocorax	Red-billed chough (*Pyrrhocorax pyrrhocorax*)
P. pica	Eurasian magpie (*Pica pica*)
Sturnus sp.	Starlings (*Sturnus* sp.) referred as common starling (*Sturnus vulgaris*) and spotless vulgaris (*Sturnus unicolor*)
C. palumbus	Common wood pigeon (*Columba palumbus*)
C. livia	Rock dove (*Columba livia*)
C. oenas	Stock dove (*Columba livia*)
S. decaocto	Eurasian collared dove (*Streptopelia decaocto*)
A. ibis	Cattle egret (*Ardea ibis*)

P. falcinellus	Glossy ibis (*Plegadis falcinellus*)
P. carbo	Great cormorant (*Phalacrocorax carbo*)
C. ciconia	White stork (*Ciconia ciconia*)
M. milvus	Red kite (*Milvus milvus*)
C. aeruginosus	Western marsh harrier (*Circus aeruginosus*)
P. krameri	Rose-ringed parakeet (*Psittacula krameri*)
