# Supplementary material for: Large-scale interspecific associations and ecological context shape communal roosts of Western jackdaw (Coloeus monedula)
Source: PLoS One. 2026 May 20;21(5):e0346626. doi: 10.1371/journal.pone.0346626 (PMC13189308; doi:10.1371/journal.pone.0346626)
Supplement: S2 Table — This table presents all bird species observed co-occurring with jackdaws in winter communal roosts. For each species, the table includes: the scientific names; the percentage of shared roosts in which the species was present, and with the percentage of occupied roosts by substrate type (Tree / Wetland / Other); the mean specific abundance ± standard deviation (SD) within the roosts they occupied each species, and the mean ± SD of the species’ prevalence within jackdaw roosts (i.e., its percentage contribution to total roost size); the mean ± SD species richness in jackdaw roosts where the species occurred; the mean ± SD number of jackdaws present in those roosts, and the mean ± SD of the jackdaw’s proportional contribution to roost size. Species marked with an asterisk (*) are those that regularly formed roosts and were included in the analyses. Species detected in only six or fewer roosts were excluded from analyses. Variable names and details follow the definitions in S1 Table. (PDF) [file pone.0346626.s002.pdf]

S2 Table. Species recorded sharing winter communal roosts with the jackdaw (*Coloeus monedula*). This table presents all bird species observed co-occurring with jackdaws in winter communal roosts. For each species, the table includes: the scientific names; the percentage of shared roosts in which the species was present, and with the percentage of occupied roosts by substrate type (Tree / Wetland / Other); the mean specific abundance ± standard deviation (SD) within the roosts they occupied each species, and the mean ± SD of the species' prevalence within jackdaw roosts (i.e., its percentage contribution to total roost size); the mean ± SD species richness in jackdaw roosts where the species occurred; the mean ± SD number of jackdaws present in those roosts, and the mean ± SD of the jackdaw's proportional contribution to roost size. Species marked with an asterisk (*) are those that regularly formed roosts and were included in the analyses. Species detected in only six or fewer roosts were excluded from analyses. Variable names and details follow the definitions in Table S1.

**Species**	**% of shared roosts tree / wetland / other**	**Mean abundance ±SD (% of total roost size ±SD)**	**Mean richness ± SD**	**Mean jackdaw abundance ± SD (% of total roost size ± SD)**
*Corvus corone**	13.25 (13.29/1.92/0)	214 ± 329 (31.69 ± 31.59)	2.77 ± 1.02	619 ± 1,329 (56.26 ± 31.27)
*Corvus corax**	7.23 (6.33/3.85/0)	54 ± 93 (15.77 ± 23.05)	2.97 ± 0.93	152 ± 128 (56.33 ± 22.14)
Corvus frugilegus	1.81 (1.90/0/0)	1,300 ± 841 (62.80 ± 19.05)	2.67 ± 0.58	508 ± 118 (30.75 ± 11.96)
*Pica pica**	36.75 (32.28/19.23/0)	93 ± 123 (21.26 ± 18.62%)	3.33 ± 0.98	357 ± 520 (51.98 ± 27.26)
Pyrrhocorax pyrrhocorax	2.41 (0.88/0/13.64)	22.00 ± 31 (28.19 ± 38.64)	2.25 ± 0.50	73 ± 88 (65.37 ± 33.20)
Sturnus sp*	28.31 (21.52/19.23/13.64)	2,628 ± 6,220 (51.06 ± 27.25)	3.23 ± 0.87	545 ± 625 (33.77 ± 20.04)
*Columba palumbus**	19.28 (18.99/3.85/0)	311 ± 581 (21.12 ± 19.72)	3.31 ± 1.03	806 ± 1,044 (52.81 ± 27.27)
Columba oenas	0.60 (0.63/0/0)	72 ± 0 (12.41 ± 0)	3.00 ± 0.00	258 ± 0 (44.48 ± 0)
Columba livia	1.81 (0/0/13.64)	29 ± 0 (38.27 ± 43.84%)	3.00 ± 1.00	83 ± 85 (46.03 ± 30.85)
Streptopelia decaocto	1.20 (1.27/0/0)	185 ± 163 (45.61 ± 44.28)	4.00 ± 2.83	95 ± 104 (15.54 ± 10.66)
*Ardea ibis**	33.13 (15.19/59.62/0)	512 ± 870 (36.65 ± 30.65)	2.82 ± 0.92	771 ± 1,100 (47.69 ± 32.15)
*Plegadis falcinellus**	5.42 (4.43/3.85/0)	16 ± 21 (7.48 ± 9.10)	3.67 ± 1.00	353 ± 574 (24.81 ± 28.49)
*Phalacrocorax carbo**	9.64 (3.80/15.38/9.09)	74 ± 84 (8.40 ± 8.95)	3.19 ± 1.05	855 ± 1,235 (61.65 ± 30.87)
Ciconia ciconia	1.81 (1.27/1.92/0)	50 ± 0 (4.35 ± 0.00)	3.00 ± 0.00	265 ± 378 (60.87 ± 0.00)

*Milvus milvus**	3.61<br>(3.80/0/0)	74 ± 89<br>(14.00 ± 16.50%)	4.00 ± 0.89	251 ± 121<br>(52.51 ± 21.27)
Circus aeruginosus	1.20<br>(0 /3.85/0)	56 ± 70<br>(1.40 ± 1.24)	3.00 ± 1.41	772 ± 509<br>(54.20 ± 64.03)
Psittacula krameri	1.20<br>(1.27/0/0)	43 ± 10<br>(8.82 ± 5.27)	4.50 ± 0.71	419 ± 261<br>(71.44 ± 14.28)
