# Supplementary material for: Large-scale interspecific associations and ecological context shape communal roosts of Western jackdaw (Coloeus monedula)
Source: PLoS One. 2026 May 20;21(5):e0346626. doi: 10.1371/journal.pone.0346626 (PMC13189308; doi:10.1371/journal.pone.0346626)
Supplement: S3 Table — The null model was included in our set of models. df: degrees of freedom; AICc: Akaike information criterion corrected for small sample sizes; ΔAICc: difference between the AICc of model i and that of the best model (i.e., the model with the lowest AICc); w: Akaike weight. (PDF) [file pone.0346626.s003.pdf]

S3 Table. GLM (binomial error) model selection of western jackdaw (*Coloeus monedula*) roost sharing at both scales (500 m and 20 km) (ΔAICc < 2). The null model was included in our set of models. df: degrees of freedom; AICc: Akaike information criterion corrected for small sample sizes; ΔAICc: difference between the AICc of model i and that of the best model (i.e. the model with the lowest AICc); w: Akaike weight.

Models	df	ΔAICc	w
Substrate + *C. monedula* + Distance to landfills + Temperature500m + Elevation500m + Urban500m + Mosaic crops500m + Forests20km	10	0.00	0.44
Substrate + *C. monedula* + Distance to landfills + Temperature500m + Elevation500m + Urban500m + Mosaic crops500m + Forests20km + Shrublands20km	11	0.59	0.33
Substrate + *C. monedula* + Distance to landfills + Temperature500m + Elevation500m + Urban500m + Mosaic crops500m + Irrigated crops20km + Forests20km	11	1.38	0.22
