# Supplementary material for: Large-scale interspecific associations and ecological context shape communal roosts of Western jackdaw (Coloeus monedula)
Source: PLoS One. 2026 May 20;21(5):e0346626. doi: 10.1371/journal.pone.0346626 (PMC13189308; doi:10.1371/journal.pone.0346626)
Supplement: S4 Table — Estimates and 95% confidence intervals were assessed. In bold, effects that received significant support (i.e., the 95% CI does not overlap zero). (PDF) [file pone.0346626.s004.pdf]

S4 Table. Alternative binomial GLM models explaining western jackdaw (*Coloeus monedula*) roost sharing in relation to environmental variables measured at two spatial scales (500 m and 20 km), with model support defined by ΔAIC < 2. Estimates and 95% confidence intervals were assessed. In bold, effects that received significant support (i.e. the 95% CI does not overlap zero).

Variable	Estimate	2.5% CI	97.5% CI
**Intercept**	1.82	1.14	2.50
Roost substrate: Wetland	0.37	-0.69	1.42
**Roost substrate: Others**	-1.91	-3.04	-0.78
C. monedula abundance	2.33	0.86	3.80
**Distance to landfills**	0.45	0.03	0.87
**Temperature500m**	-0.44	-0.82	-0.06
**Elevation500m**	-0.53	-0.92	-0.13
**Urban500m**	0.42	0.02	0.82
**Mosaic crops500m**	-0.41	-0.73	-0.09
**Forests20km**	-0.47	-0.86	-0.08
**Intercept**	1.81	1.13	2.49
Roost substrate: Wetland	0.35	-0.71	1.41
**Roost substrate: Others**	-1.86	-2.99	-0.74
C. monedula abundance	2.28	0.82	3.74
**Distance to landfills**	0.51	0.08	0.94
**Temperature500m**	-0.45	-0.83	-0.07
**Elevation500m**	-0.52	-0.91	-0.13
**Urban500m**	0.45	0.05	0.85
**Mosaic crops500m**	-0.37	-0.70	-0.04
**Forests20km**	-0.45	-0.84	-0.07
Shrublands20km	-0.24	-0.61	0.13
**Intercept**	1.79	1.11	2.47
Roost substrate: Wetland	0.44	-0.63	1.52
**Roost substrate: Others**	-1.83	-2.96	-0.70
C. monedula	2.24	0.78	3.70
**Distance to landfills**	0.48	0.06	0.90

**Temperature500m**	-0.44	-0.82	-0.06
**Elevation500m**	-0.48	-0.88	-0.08
**Urban500m**	0.45	0.04	0.85
**Mosaic crops500m**	-0.39	-0.72	-0.07
Irrigated crops20km	0.21	-0.25	0.66
**Forests20km**	-0.45	-0.83	-0.06
