# Supplementary material for: Large-scale interspecific associations and ecological context shape communal roosts of Western jackdaw (Coloeus monedula)
Source: PLoS One. 2026 May 20;21(5):e0346626. doi: 10.1371/journal.pone.0346626 (PMC13189308; doi:10.1371/journal.pone.0346626)
Supplement: S5 Table — The null model was included in our set of models. df: degrees of freedom; ΔAICc: difference between the AICc (Akaike information criterion corrected for small sample sizes) of model i and that of the best model (i.e., the model with the lowest AICc); w: Akaike weight. (PDF) [file pone.0346626.s005.pdf]

S5 Table. GLM (log-normal error) model selection of western jackdaw (*Coloeus monedula*) roost size in relation to the presence/absence of co-roosting species in the Iberian Peninsula (ΔAICc < 2). The null model was included in our set of models. df: degrees of freedom; ΔAICc: difference between the AICc (Akaike information criterion corrected for small sample sizes) of model i and that of the best model (i.e. the model with the lowest AICc); w: Akaike weight.

Models	df	ΔAICc	w
P. falcinellus + *P. carbo* + *A. ibis* + *Sturnus* sp. + *C. palumbus* + *C. corone*	8	0.00	0.12
P. falcinellus + *P. carbo* + *A. ibis* + *Sturnus* sp. + *C. palumbus*	7	0.53	0.09
P. falcinellus + *P. carbo* + *A. ibis* + *Sturnus* sp. + *C. palumbus* + *P. pica*	8	0.75	0.08
C. corax + *P. falcinellus* + *P. carbo* + *A. ibis* + *Sturnus* sp. + *C. palumbus* + *C. corone*	9	1.04	0.07
C. corax + *Mmilvus* + *P. falcinellus* + *P. carbo* + *A. ibis* + *Sturnus* sp. + *C. palumbus* + *P. pica*	10	1.05	0.07
P. falcinellus + *A. ibis* + *Sturnus* sp. + *C. palumbus* + *P. pica*	7	1.23	0.06
C. corax + *Mmilvus* + *P. falcinellus* + *A. ibis* + *Sturnus* sp. + *C. palumbus* + *P. pica*	9	1.24	0.06
C. corax + *M. milvus* + *P. falcinellus* + *P. carbo* + *A. ibis* + *Sturnus* sp. + *C. palumbus*	9	1.39	0.06
P. falcinellus + *P. carbo* + *A. ibis* + *Sturnus* sp. + *C. palumbus* + *C. corone* + *P. pica*	9	1.44	0.06
P. falcinellus + *A. ibis* + *Sturnus* sp. + *C. palumbus*	6	1.47	0.06
P. falcinellus + *A. ibis* + *Sturnus* sp. + *C. palumbus* + *C. corone*	7	1.56	0.06
C. corax + *P. falcinellus* + *P. carbo* + *A. ibis* + *Sturnus* sp. + *C. palumbus* + *P. pica*	9	1.58	0.05
C. corax + *P. falcinellus* + *A. ibis* + *Sturnus* sp. + *C. palumbus* + *P. pica*	8	1.72	0.05
C. corax + *P. falcinellus* + *P. carbo* + *A. ibis* + *Sturnus* sp. + *C. palumbus*	8	1.78	0.05
C. corax + *M. milvus* + *P. falcinellus* + *P. carbo* + *A. ibis* + *Sturnus* sp. + *C. palumbus* + *C. corone*	10	1.80	0.05
