# Supplementary material for: Large-scale interspecific associations and ecological context shape communal roosts of Western jackdaw (Coloeus monedula)
Source: PLoS One. 2026 May 20;21(5):e0346626. doi: 10.1371/journal.pone.0346626 (PMC13189308; doi:10.1371/journal.pone.0346626)
Supplement: S6 Table — Estimates and 95% confidence intervals are shown. In bold, effects that received significant support (i.e., the 95% CI does not overlap zero). (PDF) [file pone.0346626.s006.pdf]

S6 Table. Alternative log-normal GLM models explaining western jackdaw (*Coloeus monedula*) roost size in relation to the presence/absence of co-roosting species in the Iberian Peninsula, with model support defined by ΔAIC < 2. Estimates and 95% confidence intervals are shown. In bold, effects that received significant support (i.e. the 95% CI does not overlap zero).

Variable	Estimate	2.5% CI	97.5% CI
Intercept	4.72	4.33	5.10
P. falcinellus	-1.42	-2.42	-0.43
P. carbo	0.74	-0.01	1.48
A. ibis	0.84	0.32	1.36
***Sturnus* sp.**	0.59	0.10	1.08
C. palumbus	0.82	0.26	1.37
C. corone	0.58	-0.10	1.26
Intercept	4.88	4.55	5.21
P. falcinellus	-1.42	-2.42	-0.40
P. carbo	0.67	-0.07	1.42
A. ibis	0.71	0.21	1.21
***Sturnus* sp.**	0.50	0.01	0.98
C. palumbus	0.73	0.18	1.28
Intercept	5.04	4.65	5.43
P. falcinellus	-1.43	-2.43	-0.43
P. carbo	0.62	-0.12	1.37
A. ibis	0.61	0.08	1.13
***Sturnus* sp.**	0.53	0.05	1.01
C. palumbus	0.76	0.21	1.31
P. pica	-0.34	-0.82	0.13
Intercept	4.78	4.39	5.18
P. falcinellus	-1.42	-2.42	-0.42
P. carbo	0.71	-0.03	1.45
A. ibis	0.79	0.26	1.32
***Sturnus* sp.**	0.55	0.06	1.04
C. palumbus	0.79	0.24	1.35

C. corone	0.60	-0.08	1.28
C. corax	-0.47	-1.32	0.37
Intercept	5.12	4.70	5.54
P. falcinellus	-1.43	-2.41	-0.44
P. carbo	0.59	-0.15	1.32
A. ibis	0.54	0.01	1.07
Sturnus	0.48	-0.004	0.96
C. palumbus	0.77	0.22	1.31
P. pica	-0.39	-0.87	0.08
C. corax	-1.00	-2.01	0.02
M. milvus	1.17	-0.20	2.54
Intercept	5.10	4.71	5.49
P. falcinellus	-1.48	-2.49	-0.47
A. ibis	0.67	0.16	1.19
***Sturnus* sp.**	0.50	0.01	0.98
C. palumbus	0.76	0.20	1.31
P. pica	-0.38	-0.86	0.10
Intercept	5.19	4.78	5.61
P. falcinellus	-1.47	-2.47	-0.48
A. ibis	0.60	0.07	1.13
Sturnus sp.	0.44	-0.04	0.92
C. palumbus	0.76	0.21	1.31
P. pica	-0.43	-0.91	0.04
C. corax	-1.05	-2.07	-0.03
M. milvus	1.17	-0.21	2.55
Intercept	4.93	4.58	5.29
P. falcinellus	-1.41	-2.41	-0.41
P. carbo	0.65	-0.08	1.39
A. ibis	0.67	0.17	1.18
Sturnus sp.	0.45	-0.03	0.94
C. palumbus	0.73	0.19	1.28
C. corax	-0.89	-1.90	0.13
M. milvus	1.15	-0.23	2.53

Intercept	4.85	4.37	5.34
P. falcinellus	-1.43	-2.43	-0.44
P. carbo	0.69	-0.05	1.44
A. ibis	0.74	0.18	1.31
***Sturnus* sp.**	0.59	0.10	1.08
C. palumbus	0.82	0.27	1.38
C. corone	0.46	-0.26	1.18
P. pica	-0.23	-0.74	0.28
Intercept	4.93	4.61	5.26
P. falcinellus	-1.47	-2.48	-0.45
A. ibis	0.80	0.30	1.30
***Sturnus* sp.**	0.46	-0.02	0.95
C. palumbus	0.72	0.17	1.28
Intercept	4.79	4.41	5.17
P. falcinellus	-1.48	-2.49	-0.47
A. ibis	0.92	0.40	1.44
***Sturnus* sp.**	0.54	0.04	1.03
C. palumbus	0.80	0.24	1.36
C. corone	0.51	-0.18	1.19
Intercept	5.14	4.71	5.57
P. falcinellus	-1.43	-2.42	-0.43
P. carbo	0.59	-0.16	1.59
A. ibis	0.53	-0.02	1.53
***Sturnus* sp.**	0.49	0.002	1.49
C. palumbus	0.73	0.18	1.73
P. pica	-0.38	-0.86	0.38
C. corax	-0.52	-1.38	0.34
Intercept	5.21	4.79	5.62
P. falcinellus	-1.47	-2.48	-0.47
A. ibis	0.59	0.06	1.12
Sturnus sp.	0.45	-0.03	0.94
C. palumbus	0.72	0.17	1.28
P. pica	-0.42	-0.90	0.06

C. corax	-0.58	-1.44	0.28
Intercept	4.95	4.60	5.30
P. falcinellus	-1.41	-2.42	-0.41
P. carbo	0.65	-0.10	1.39
A. ibis	0.66	0.15	1.17
Sturnus sp.	0.46	-0.02	0.95
C. palumbus	0.70	0.15	1.25
C. corax	-0.43	-1.28	0.43
Intercept	4.80	4.41	5.20
P. falcinellus	-1.42	-2.41	-0.43
P. carbo	0.70	-0.03	1.44
A. ibis	0.78	0.25	1.30
***Sturnus* sp.**	0.53	0.03	1.02
C. palumbus	0.80	0.25	1.35
C. corone	0.49	-0.21	1.19
C. corax	-0.82	-1.84	0.20
M. milvus	0.89	-0.54	2.31
