# Supplementary material for: Large-scale interspecific associations and ecological context shape communal roosts of Western jackdaw (Coloeus monedula)
Source: PLoS One. 2026 May 20;21(5):e0346626. doi: 10.1371/journal.pone.0346626 (PMC13189308; doi:10.1371/journal.pone.0346626)
Supplement: S7 Table — The null model was included in our set of models. df: degrees of freedom; ΔAICc: difference between the AICc (Akaike information criterion corrected for small sample sizes) of model i and that of the best model (i.e., the model with the lowest AICc); w: Akaike weight. (PDF) [file pone.0346626.s007.pdf]

S7 Table. GLM (log-normal error) model selection of western jackdaw (*Coloeus monedula*) roost size in relation to the specific abundances of co-roosting species in the Iberian Peninsula (ΔAICc < 2). The null model was included in our set of models. df: degrees of freedom; ΔAICc: difference between the AICc (Akaike information criterion corrected for small sample sizes) of model i and that of the best model (i.e. the model with the lowest AICc); w: Akaike weight.

Models	df	ΔAICc	w
Het_abundance + *Sturnus* sp. + *P. falcinellus*	5	0.00	0.23
Het_abundance + *Sturnus* sp. + *P. falcinellus* + *C. palumbus*	6	1.17	0.13
A. ibis + *C. palumbus* + *P. falcinellus*	5	1.20	0.13
Het_abundance + *Sturnus* sp.+ *P. falcinellus* + *C. corone*	6	1.20	0.13
Het_abundance + *Sturnus* sp. + *P. falcinellus* + *P. pica*	6	1.69	0.10
Het_abundance + Richness + *Sturnus* sp. + *P. falcinellus*	6	1.82	0.09
Het_abundance + *A. ibis* + *Sturnus* sp. + *P. falcinellus*	6	1.87	0.09
Het_abundance + *A. ibis* + *P. falcinellus* + *C. palumbus*	6	1.91	0.09
