# Supplementary material for: Large-scale interspecific associations and ecological context shape communal roosts of Western jackdaw (Coloeus monedula)
Source: PLoS One. 2026 May 20;21(5):e0346626. doi: 10.1371/journal.pone.0346626 (PMC13189308; doi:10.1371/journal.pone.0346626)
Supplement: S8 Table — Estimates and 95% confidence intervals are shown. In bold, effects that received significant support (i.e., the 95% CI does not overlap zero). (PDF) [file pone.0346626.s008.pdf]

S8 Table. Alternative log-normal GLM models explaining western jackdaw (*Coloeus monedula*) roost size in relation to the specific abundances of co-roosting species in the Iberian Peninsula, with model support defined by ΔAIC < 2. Estimates and 95% confidence intervals are shown. In bold, effects that received significant support (i.e. the 95% CI does not overlap zero).

**Variable**	**Estimate**	**2.5% CI**	**97.5% CI**
Intercept	5.37	5.14	5.61
**Het_abundance**	1.57	0.57	2.57
P. falcinellus	-0.29	-0.49	-0.10
***Sturnus* sp.**	-1.40	-2.39	-0.40
Intercept	5.37	5.14	5.61
**Het_abundance**	1.35	0.27	2.43
P. falcinellus	-0.29	-0.48	-0.10
***Sturnus* sp.**	-1.19	-2.26	-0.12
C. palumbus	0.11	-0.10	0.31
Intercept	5.40	5.17	5.63
A. ibis	0.23	0.04	0.43
P. falcinellus	-0.30	-0.49	-0.11
C. palumbus	0.22	0.03	0.41
Intercept	5.38	5.15	5.61
**Het_abundance**	1.64	0.63	2.65
P. falcinellus	-0.30	-0.49	-0.11
***Sturnus* sp.**	-1.47	-2.47	-0.47
C. corone	-0.10	-0.29	0.10
Intercept	5.36	5.13	5.60
**Het_abundance**	1.58	0.58	2.58
P. falcinellus	-0.29	-0.48	-0.10
***Sturnus* sp.**	-1.41	-2.40	-0.42
P. pica	0.07	-0.13	0.27
Intercept	5.33	5.06	5.60
**Het_abundance**	1.56	0.57	2.56
Richness	0.09	-0.20	0.38

P. falcinellus	-0.30	-0.49	-0.11
***Sturnus* sp.**	-1.40	-2.39	-0.41
Intercept	5.37	5.14	5.60
**Het_abundance**	1.95	0.27	3.64
A. ibis	-0.09	-0.42	0.23
P. falcinellus	-0.29	-0.48	-0.10
***Sturnus* sp.**	-1.76	-3.39	-0.14
Intercept	5.39	5.16	5.62
Het_abundance	0.12	-0.08	0.32
A. ibis	0.20	0.002	0.40
P. falcinellus	-0.30	-0.49	-0.10
C. palumbus	0.21	0.01	0.40
