# Supplementary material for: Large-scale interspecific associations and ecological context shape communal roosts of Western jackdaw (Coloeus monedula)
Source: PLoS One. 2026 May 20;21(5):e0346626. doi: 10.1371/journal.pone.0346626 (PMC13189308; doi:10.1371/journal.pone.0346626)
Supplement: S9 Table — The null model was included in our set of models. df: degrees of freedom; AICc: Akaike information criterion corrected for small sample sizes; ΔAICc: difference between the AICc of model i and that of the best model (i.e., the model with the lowest AICc); w: Akaike weight. (PDF) [file pone.0346626.s009.pdf]

S9 Table. GLM (log-normal error) model selection of western jackdaw (*Coloeus monedula*) roost size in relation to the specific abundances of co-roosting species in the Iberian Peninsula, subdivided into the different substrates (ΔAICc < 2). The null model was included in our set of models. df: degrees of freedom; AICc: Akaike information criterion corrected for small sample sizes; ΔAICc: difference between the AICc of model i and that of the best model (i.e. the model with the lowest AICc); w: Akaike weight.

**Models**	**df**	**ΔAICc**	**w**
**Tree**			
Het_abundance + *P. falcinellus* + *C. palumbus*	5	0.00	0.16
P. falcinellus + *C. palumbus*	4	0.09	0.15
P. falcinellus + *C. palumbus* + *Sturnus* sp.	5	0.30	0.13
Het_abundance + *P. falcinellus* + *Sturnus* sp.	5	0.89	0.10
Het_abundance + *P. falcinellus* + *C. palumbus* + *Sturnus* sp.	6	1.46	0.08
P. falcinellus + *C. palumbus* + *A. ibis*	5	1.50	0.07
Richness + *P. falcinellus* + *C. palumbus*	5	1.72	0.07
Het_abundance + *P. falcinellus* + *Sturnus* sp. + *P. carbo*	6	1.76	0.06
Het_abundance + *P. falcinellus* + *C. palumbus* + *P. pica*	6	1.87	0.06
P. falcinellus + *C. palumbus* + *P. pica*	5	1.92	0.06
Het_abundance + Richness + *P. falcinellus* + *C. palumbus*	6	1.96	0.06
**Wetland**			
P. falcinellus + *C. corax* + *C. corone*	5	0.00	0.24
P. falcinellus + *C. corax* + *C. corone* + *P. carbo*	6	1.17	0.14
Het_abundance + *P. falcinellus* + *C. corax* + *C. corone*	6	1.47	0.12
P. falcinellus + *C. corax*	4	1.50	0.12
Het_abundance + *P. falcinellus* + *C. corax* + *C. corone* + *P. carbo*	7	1.77	0.10
Het_abundance + *P. falcinellus* + *C. corone* + *P. carbo*	6	1.83	0.10
P. falcinellus + *C. corax* + *C. corone* + *A. ibis*	6	1.87	0.10
P. falcinellus + *C. corone*	4	1.99	0.09
**Others**			
NULL	2	0.00	1.00
