# Supplementary material for: Large-scale interspecific associations and ecological context shape communal roosts of Western jackdaw (Coloeus monedula)
Source: PLoS One. 2026 May 20;21(5):e0346626. doi: 10.1371/journal.pone.0346626 (PMC13189308; doi:10.1371/journal.pone.0346626)
Supplement: S10 Table — Estimates and 95% confidence intervals were assessed. In bold, effects that received significant support (i.e., the 95% CI does not overlap zero). (*) indicates the variables that were significant in some alternative models but not in the average model. Variances explained: Tree = 14.06%; Wetland = 39.70%; Other = 0.0%. (PDF) [file pone.0346626.s010.pdf]

S10 Table. Model averaging of all alternative log-normal GLM models (ΔAICc < 2) of roosting jackdaw roost size in relation to the specific abundances of co-roosting species in the Iberian Peninsula by roost substrate types. Estimates and 95% confidence intervals were assessed. In bold, effects that received significant support (i.e. the 95% CI does not overlap zero). (*) indicates the variables that were significant in some alternative models but not in the average model. Variances explained: Tree =14.06%; Wetland=39.70%; Other =0.0%.

Variable	Estimate	2.5% CI	97.5% CI
**Tree**			
Intercept	5.31	5.04	5.59
Het_abundance (*)	0.79	-1.04	2.62
Richness	0.10	-0.20	0.40
P. falcinellus	-0.25	-0.43	-0.06
Sturnus sp. (*)	-0.83	-2.83	1.16
A. ibis	0.20	-0.24	0.64
C. palumbus	0.22	0.02	0.41
P. pica	0.06	-0.14	0.27
P. carbo	-0.13	-0.36	0.09
**Wetland**			
Intercept	7.14	3.56	10.72
Het_abundance	0.77	-0.10	1.83
C. corone	16.52	1.51	31.53
C. corax	-13.93	-27.52	-0.34
A. ibis	0.10	-0.10	0.30
P. carbo	0.30	-0.09	0.68
P. falcinellus	-1.15	-1.92	0.39
**Other**			
Intercept	3.52	2.61	4.42
