# Supplementary material for: Large-scale interspecific associations and ecological context shape communal roosts of Western jackdaw (Coloeus monedula)
Source: PLoS One. 2026 May 20;21(5):e0346626. doi: 10.1371/journal.pone.0346626 (PMC13189308; doi:10.1371/journal.pone.0346626)
Supplement: S11 Table — Estimates and 95% confidence intervals were assessed. In bold, effects that received significant support (i.e., the 95% CI does not overlap zero). (PDF) [file pone.0346626.s011.pdf]

S11 Table. Alternative log-normal GLM models explaining western jackdaw (*Coloeus monedula*) roost size in relation to the specific abundances of co-roosting species in the Iberian Peninsula, subdivided into the different substrates, with model support defined by ΔAIC < 2. Estimates and 95% confidence intervals were assessed. In bold, effects that received significant support (i.e. the 95% CI does not overlap zero).

Variable	Estimate	2.5% CI	97.5% CI
**Tree**			
Intercept	5.31	5.06	5.57
Het_abundance	0.14	-0.04	0.32
P. falcinellus	-0.25	-0.43	-0.06
C. palumbus	0.22	0.04	0.40
Intercept	5.34	5.08	5.59
P. falcinellus	-0.25	-0.43	-0.07
C. palumbus	0.22	0.04	0.41
Intercept	5.32	5.06	5.57
P. falcinellus	-0.25	-0.43	-0.06
C. palumbus	0.23	0.05	0.41
Sturnus sp.	0.13	-0.05	-0.31
Intercept	5.32	5.07	5.58
Het_abundance	1.66	-0.27	3.05
P. falcinellus	-0.25	-0.44	-0.07
***Sturnus* sp.**	-1.50	-2.87	-0.12
Intercept	5.31	5.05	5.57
Het_abundance	0.94	-0.82	2.70
P. falcinellus	-0.25	-0.43	-0.07
C. palumbus	0.15	-0.08	0.38
Sturnus sp.	-0.79	-2.52	0.94
Intercept	5.35	5.09	5.61
P. falcinellus	-0.26	-0.44	-0.07
C. palumbus	0.23	0.05	0.41
A. ibis	0.20	-0.24	0.63
Intercept	5.27	4.96	5.58

Richness	0.12	-0.18	0.41
P. falcinellus	-0.26	-0.44	-0.07
C. palumbus	0.22	0.04	0.40
Intercept	5.06	5.06	5.57
**Het_abundance**	2.04	0.52	3.57
P. falcinellus	-0.26	-0.44	-0.07
***Sturnus* sp.**	-1.86	-3.35	-0.37
P. carbo	-0.13	-0.36	0.09
Intercept	5.30	5.04	5.56
Het_abundance	0.14	-0.04	0.32
P. falcinellus	-0.24	-0.42	-0.06
C. palumbus	0.22	0.04	0.40
P. pica	0.06	-0.14	0.27
Intercept	5.32	5.06	5.58
P. falcinellus	-0.25	-0.43	-0.06
C. palumbus	0.23	0.05	0.41
P. pica	0.07	-0.14	0.27
Intercept	5.27	4.96	5.57
Het_abundance	0.13	-0.05	0.32
Richness	0.08	-0.21	0.38
P. falcinellus	-0.25	-0.43	-0.07
C. palumbus	0.21	0.03	0.39
**Wetland**			
Intercept	6.96	4.17	9.75
P. falcinellus	-1.20	-1.92	-0.48
C. corax	-14.71	-27.59	-1.84
C. corone	15.41	1.11	29.72
Intercept	7.13	4.39	9.87
P. falcinellus	-1.16	-1.87	-0.45
C. corax	-13.68	-26.34	-1.02
C. corone	16.24	2.22	30.26
P. carbo	0.24	-0.11	0.60
Intercept	7.26	4.48	10.05

Het_abundance	0.60	-0.37	1.56
P. falcinellus	-1.14	-1.86	-0.43
C. corax	-13.35	-26.16	-0.53
C. corone	16.54	2.39	30.68
Intercept	4.37	2.87	5.87
P. falcinellus	-1.22	-1.99	-0.46
C. corax	-15.30	-28.93	-1.66
Intercept	7.58	4.88	10.28
Het_abundance	0.79	-0.17	1.74
P. falcinellus	-1.07	-1.77	-0.38
C. corax	-11.59	-24.08	0.90
C. corone	17.95	4.25	31.64
P. carbo	0.31	-0.05	0.66
Intercept	8.95	6.58	11.31
Het_abundance	0.97	-0.01	1.95
P. falcinellus	-1.02	-1.74	-0.30
C. corone	18.98	4.71	33.25
P. carbo	0.36	-0.003	0.73
Intercept	7.09	4.33	9.86
P. falcinellus	-1.17	-1.88	-0.45
C. corax	-13.84	-26.64	-1.04
C. corone	16.11	1.95	30.28
A. ibis	0.10	-0.09	0.30
Intercept	8.63	6.09	11.17
P. falcinellus	-1.16	-1.94	-0.39
C. corone	16.10	0.85	31.36
**Other**			
Intercept	3.52	2.61	4.42
