# Supplementary material for: Large-scale interspecific associations and ecological context shape communal roosts of Western jackdaw (Coloeus monedula)
Source: PLoS One. 2026 May 20;21(5):e0346626. doi: 10.1371/journal.pone.0346626 (PMC13189308; doi:10.1371/journal.pone.0346626)
Supplement: S12 Table — Estimates and 95% confidence intervals were assessed. In bold, effects that received significant support (i.e., the 95% CI does not overlap zero). Additionally, model error distributions and the proportion of variance explained (R²) were evaluated for each model. (PDF) [file pone.0346626.s012.pdf]

S12 Table. GLM models of univariant jackdaw roost size by specific abundances of co-roosting species (ΔAIC < 2). Estimates and 95% confidence intervals were assessed. In bold, effects that received significant support (i.e. the 95% CI does not overlap zero). Additionally, model error distributions and the proportion of variance explained (R^2) were evaluated for each model.

Variable	Estimate	SE	2.5% CI	97.5% CI	Distribution	Expl. Var.
Intercept	5.51	0.37	4.80	6.23	Log-normal	2.29%
C. corone	-0.06	0.11	-0.27	0.14		
Intercept	5.22	0.17	4.88	5.55	Log-normal	15.67%
P. pica	0.45	0.17	0.11	0.79		
Intercept	4.80	0.31	4.20	5.40	Log-normal	26.04%
C. corax	0.52	0.33	-0.13	1.16		
Intercept	6.16	0.17	5.82	6.49	Quadratic	20.41%
***Sturnus* sp.**	-1.73	0.78	-3.25	-0.21		
Intercept	6.83	0.23	6.37	7.29	Gamma	1.42%
C. palumbus	0.18	0.30	-0.40	0.77		
Intercept	5.86	0.29	5.29	6.44	Log-normal	3.55%
A. ibis	0.32	0.30	-0.26	0.90		
Intercept	3.78	0.42	2.96	4.59	Log-normal	34.65%
P. falcinellus	-0.80	0.45	-1.69	0.08		
Intercept	6.06	0.31	5.45	6.67	Log-normal	0.27%
P. carbo	-0.06	0.32	-0.69	0.57		
