# Supplementary material for: Large-scale interspecific associations and ecological context shape communal roosts of Western jackdaw (Coloeus monedula)
Source: PLoS One. 2026 May 20;21(5):e0346626. doi: 10.1371/journal.pone.0346626 (PMC13189308; doi:10.1371/journal.pone.0346626)
Supplement: S13 Table — (PDF) [file pone.0346626.s013.pdf]

S13 Table. Summary of dominant species across communal roosts, including the percentage of roosts in which each species was dominant when each species is present, average jackdaw abundance, and average species-specific roost size when each species dominates or not the shared communal roost.

		Coloeus monedula		**Associated species**	
**Species**	**% roost dominance**	**dominant species roost size**	**not dominant species roost size**	**dominant species roost size**	**not dominant species roost size**
C. monedula	55.10	842 ± 1,244	326 ± 442	-	-
C. corax	10.00	28 ± 0	189 ± 128	74 ± 0	52 ± 99
C. corone	22.73	127 ± 126.2	764 ± 1,489	594 ± 532	102 ± 114
C. frugilegus	66.67	575 ± 35	375 ± 0	1,775 ± 248	350 ± 0
P. pica	15.63	113 ± 173	376 ± 556	178 ± 242	77 ± 80
P. pyrrhocorax	33.33	22 ± 0	120 ± 121	58 ± 0	4 ± 0
Sturnus sp.	63.89	491 ± 505	658 ± 844	3,939 ± 7,511	309 ± 549
A. ibis	36.00	317 ± 531	1,057 ± 1,296	932 ± 1,235	275 ± 446
C. livia	33.33	5 ± 0	122 ± 73.5	40 ± 0	24 ± 9
C. palumbus	16.13	304 ± 280	912 ± 1,128	865 ± 1,234	204 ± 297
S. decaocto	50.00	21 ± 0	168 ± 0	70 ± 0	300 ± 0
M. milvus	16.67	98 ± 0	281 ± 106	134 ± 0	62 ± 94
