# Supplementary material for: Large-scale interspecific associations and ecological context shape communal roosts of Western jackdaw (Coloeus monedula)
Source: PLoS One. 2026 May 20;21(5):e0346626. doi: 10.1371/journal.pone.0346626 (PMC13189308; doi:10.1371/journal.pone.0346626)
Supplement: S14 Table — The null model was included in our set of models. df: degrees of freedom; AICc: Akaike information criterion corrected for small sample sizes; ΔAICc: difference between the AICc of model i and that of the best model (i.e., the model with the lowest AICc); w: Akaike weight. (PDF) [file pone.0346626.s014.pdf]

**S14 Table.** GLM (binomial error) model selection of roosting dominance by western jackdaws (*Coloeus monedula*) (1) or other species (0) in relation to specific abundances of co-roosting species in the Iberian Peninsula (ΔAICc < 2). The null model was included in our set of models. df: degrees of freedom; AICc: Akaike information criterion corrected for small sample sizes; ΔAICc: difference between the AICc of model i and that of the best model (i.e. the model with the lowest AICc); w: Akaike weight.

| **Models** | **df** | **ΔAICc** | **w** |
|---|---|---|---|
| *P. falcinellus* + *A. ibis* + *Sturnus* sp. + *C. corone* | 5 | 0.00 | 0.19 |
| *P. falcinellus* + *A. ibis* + *Sturnus* sp. + *C. corone* + *P. pica* | 6 | 0.28 | 0.16 |
| *P. falcinellus* + *A. ibis* + *Sturnus* sp. + *C. corone* + *C. palumbus* | 6 | 0.83 | 0.12 |
| *P. falcinellus* + *A. ibis* + *Sturnus* sp. + *C. corone* + *C. palumbus* + *P. pica* | 7 | 0.87 | 0.12 |
| Richness + *P. falcinellus* + *A. ibis* + *Sturnus* sp. + *C. corone* | 6 | 0.93 | 0.12 |
| Richness + *P. falcinellus* + *A. ibis* + *Sturnus* sp. + *C. corone* + *P. pica* | 7 | 0.94 | 0.12 |
| Richness + *P. falcinellus* + *A. ibis* + *Sturnus* sp. + *C. corone* + *C. palumbus* + *P. pica* | 8 | 1.44 | 0.09 |
| Richness + *P. falcinellus* + *A. ibis* + *Sturnus* sp. + *C. corone* + *C. palumbus* | 7 | 1.72 | 0.08 |
