# Supplementary material for: Large-scale interspecific associations and ecological context shape communal roosts of Western jackdaw (Coloeus monedula)
Source: PLoS One. 2026 May 20;21(5):e0346626. doi: 10.1371/journal.pone.0346626 (PMC13189308; doi:10.1371/journal.pone.0346626)
Supplement: S15 Table — Estimates and 95% confidence intervals were assessed. In bold, effects that received significant support (i.e., the 95% CI does not overlap zero). (*) indicates the variables that were significant in some alternative models but not in the average model. The deviance explained by the averaged model is 32.72%. (PDF) [file pone.0346626.s015.pdf]

S15 Table. Model averaging of all alternative binomial GLM models (ΔAICc < 2) of roosting dominance by western jackdaws (*Coloeus monedula*) (1) or other species (0) in relation to specific abundances of co-roosting species in the Iberian Peninsula. Estimates and 95% confidence intervals were assessed. In bold, effects that received significant support (i.e. the 95% CI does not overlap zero). (*) indicates the variables that were significant in some alternative models but not in the average model. The deviance explained by the averaged model is 32.72%.

**Variable**	**Estimate**	**2.5% CI**	**97.5% CI**
Intercept	-0.81	-1.73	0.12
Richness	0.33	-0.23	0.88
P. falcinellus	-1.66	-4.03	0.70
***Sturnus* sp.**	-8.05	-13.22	-2.88
C. palumbus	-0.18	-0.49	0.13
A. ibis	-1.13	-2.06	-0.20
C. corone (*)	-0.68	-1.37	0.01
P. pica	-0.24	-0.60	0.12
