# Supplementary material for: Large-scale interspecific associations and ecological context shape communal roosts of Western jackdaw (Coloeus monedula)
Source: PLoS One. 2026 May 20;21(5):e0346626. doi: 10.1371/journal.pone.0346626 (PMC13189308; doi:10.1371/journal.pone.0346626)
Supplement: S16 Table — Estimates and 95% confidence intervals were assessed. In bold, effects that received significant support (i.e., the 95% CI does not overlap zero). (PDF) [file pone.0346626.s016.pdf]

S16 Table. Alternative binomial GLM models explaining roosting dominance by western jackdaws (*Coloeus monedula*) (1) or other species (0) in relation to specific abundances of co-roosting species in the Iberian Peninsula, with model support defined by ΔAIC < 2. Estimates and 95% confidence intervals were assessed. In bold, effects that received significant support (i.e. the 95% CI does not overlap zero).

**Variable**	**Estimate**	**2.5% CI**	**97.5% CI**
Intercept	-0.73	-1.55	0.10
P. falcinellus	-1.52	-3.73	0.70
A. ibis	-1.05	-1.92	-0.18
***Sturnus* sp.**	-7.81	-12.81	-2.81
C. corone	-0.64	-1.30	0.01
Intercept	-0.69	-1.51	0.12
P. falcinellus	-1.58	-3.84	0.67
A. ibis	-1.15	-2.07	-0.24
***Sturnus* sp.**	-7.71	-12.61	-2.81
C. corone	-0.70	-1.38	-0.02
P. pica	-0.23	-0.58	0.12
Intercept	-0.71	-1.53	0.11
P. falcinellus	-1.56	-3.80	0.68
A. ibis	-1.08	-1.96	-0.20
***Sturnus* sp.**	-7.79	-12.76	-2.82
C. corone	-0.67	-1.34	-0.01
C. palumbus	-0.17	-0.47	0.13
Intercept	-0.67	-1.48	0.14
P. falcinellus	-1.63	-3.92	0.65
A. ibis	-1.20	-2.13	-0.27
***Sturnus* sp.**	-7.69	-12.55	-2.83
C. corone	-0.74	-1.44	-0.05
C. palumbus	-0.19	-0.49	0.12
P. pica	-0.25	-0.60	0.11
Intercept	-0.98	-1.95	-0.01
Richness	0.30	-0.24	0.84
P. falcinellus	-1.73	-4.14	0.68

A. ibis	-1.08	-1.98	-0.18
***Sturnus* sp.**	-8.51	-13.85	-3.16
C. corone	-0.63	-1.30	0.04
Intercept	-0.97	-1.93	-0.01
Richness	0.34	-0.21	0.89
P. falcinellus	-1.83	-4.31	0.65
A. ibis	-1.20	-2.15	-0.25
***Sturnus* sp.**	-8.47	-13.71	-3.23
C. corone	-0.70	-1.40	-0.001
P. pica	-0.25	-0.60	0.11
Intercept	-0.95	-1.91	0.001
Richness	0.36	-0.20	0.91
P. falcinellus	-1.90	-4.42	0.62
A. ibis	-1.24	-2.21	-0.28
***Sturnus* sp.**	-8.46	-13.66	-3.26
C. corone	-0.74	-1.45	-0.02
C. palumbus	-0.20	-0.51	0.12
P. pica	-0.27	-0.63	0.10
Intercept	-0.96	-1.93	0.002
Richness	0.31	-0.23	0.85
P. falcinellus	-1.78	-4.22	0.66
A. ibis	-1.11	-2.02	-0.20
***Sturnus* sp.**	-8.50	-13.81	-3.18
C. corone	-0.66	-1.34	0.02
C. palumbus	-0.18	-0.49	0.13
