# Supplementary material for: Large-scale interspecific associations and ecological context shape communal roosts of Western jackdaw (Coloeus monedula)
Source: PLoS One. 2026 May 20;21(5):e0346626. doi: 10.1371/journal.pone.0346626 (PMC13189308; doi:10.1371/journal.pone.0346626)
Supplement: S17 Table — The null model was included in our set of models. df: degrees of freedom; AICc: Akaike information criterion corrected for small sample sizes; ΔAICc: difference between the AICc of model i and that of the best model (i.e., the model with the lowest AICc); w: Akaike weight. (PDF) [file pone.0346626.s017.pdf]

S17 Table. GLM (binomial error) model selection of roosting dominance by western jackdaws (*Coloeus monedula*) (1) or other species (0) in relation to environmental features at different scales (500 m and 20 km) in the Iberian Peninsula (ΔAICc < 2). The null model was included in our set of models. df: degrees of freedom; AICc: Akaike information criterion corrected for small sample sizes; ΔAICc: difference between the AICc of model i and that of the best model (i.e. the model with the lowest AICc); w: Akaike weight.

**Models**	**df**	**ΔAICc**	**w**
Elevation500m + Grasslands500m + Irrigated crops20km	4	0.00	0.04
Elevation500m + Urban500m	3	0.21	0.03
Elevation500m	2	0.21	0.03
Elevation500m + Irrigated crops20km	3	0.35	0.03
Elevation500m + Shrublands20km	3	0.37	0.03
Elevation500m + Grasslands500m	3	0.53	0.03
Elevation500m + Precipitation500m	3	0.60	0.03
Elevation500m + Grasslands500m + Shrublands20km	4	0.61	0.03
Elevation500m + Forests20km	3	0.62	0.03
Elevation500m + Precipitation500m + Irrigated crops20km	4	0.86	0.02
Elevation500m + Precipitation500m + Grasslands500m + Irrigated crops20km	5	0.87	0.02
Elevation500m + Urban500m + Shrublands20km	4	0.95	0.02
Elevation500m + Grasslands500m + Irrigated crops20km + Shrublands20km	5	0.96	0.02
Elevation500m + Urban500m + Irrigated crops20km	4	0.98	0.02
Elevation500m + Grasslands500m + Irrigated crops20km + AC	5	1.04	0.02
Grasslands500m + Dry crops20km	3	1.08	0.02
Elevation500m + Grasslands500m + Forests20km	4	1.11	0.02
Grasslands500m	2	1.14	0.02
Elevation500m + Precipitation500m + Urban500m	4	1.17	0.02
Elevation500m + Urban500m + Forests20km	4	1.20	0.02
Elevation500m + Precipitation500m + Grasslands500m	4	1.21	0.02
Elevation500m + Irrigated crops20km + Shrublands20km	4	1.24	0.02
Elevation500m + Irrigated crops20km + AC	4	1.33	0.02
Elevation500m + Precipitation500m + Shrublands20km	4	1.34	0.02
Elevation500m + Urban500m + AC	4	1.35	0.02

Elevation500m + Forests20km + Shrublands20km	4	1.35	0.02
Grasslands500m + Dry crops20km + Irrigated crops20km	4	1.37	0.02
Elevation500m + Urban500m + Grasslands500m	4	1.37	0.02
Elevation500m + AC	3	1.38	0.02
Elevation500m + Grasslands500m + Irrigated crops20km + Forests20km	5	1.49	0.02
Elevation500m + Grasslands500m + Dry crops20km + Irrigated crops20km	5	1.52	0.02
Elevation500m + Urban500m + Grasslands500m + Irrigated crops20km	5	1.55	0.02
Elevation500m + Irrigated crops20km + Forests20km	4	1.56	0.02
Elevation500m + Precipitation500m + Forests20km	4	1.62	0.02
Grasslands500m + Shrublands20km	3	1.63	0.02
Precipitation500m + Grasslands500m	3	1.73	0.01
Dry crops20km	2	1.75	0.01
Elevation500m + NDVI500m + Urban500m	4	1.77	0.01
Elevation500m + Grasslands500m + AC	4	1.78	0.01
Elevation500m + Grasslands500m + Shrublands20km + Forests20km	5	1.78	0.01
Elevation500m + NDVI500m + Grasslands500m + Irrigated crops20km	5	1.78	0.01
Elevation500m + Urban500m + Wetlands500m	4	1.83	0.01
Precipitation500m + Grasslands500m + Dry crops20km	4	1.85	0.01
Elevation500m + Precipitation500m + Grasslands500m + Shrublands20km	5	1.85	0.01
Elevation500m + Mosaic crops500m + Grasslands500m + Irrigated crops20km	5	1.91	0.01
Elevation500m + Dry crops20km	3	1.93	0.01
Elevation500m + Urban500m + Mosaic crops500m	4	1.93	0.01
Elevation500m + Forests20km + AC	4	1.93	0.01
Elevation500m + Urban500m + Irrigated crops20km + AC	5	1.96	0.01
Elevation500m + Precipitation500m + Urban500m + Irrigated crops20km	5	1.97	0.01
Elevation500m + Urban500m + Grasslands500m + Shrublands20km	5	1.98	0.01
Distance to landfills + Elevation500m + Grasslands500m + Irrigated crops20km	5	1.98	0.01
