# Supplementary material for: Large-scale interspecific associations and ecological context shape communal roosts of Western jackdaw (Coloeus monedula)
Source: PLoS One. 2026 May 20;21(5):e0346626. doi: 10.1371/journal.pone.0346626 (PMC13189308; doi:10.1371/journal.pone.0346626)
Supplement: S18 Table — Estimates and 95% confidence intervals were assessed. In bold, effects that received significant support (i.e., the 95% CI does not overlap zero). (*) indicates the variables that were significant in some alternative models but not in the average model. The deviance explained by the averaged model is 5.83%. (PDF) [file pone.0346626.s018.pdf]

S18 Table. Model averaging of all alternative binomial GLM models (ΔAICc < 2) of roosting dominance by western jackdaws (*Coloeus monedula*) (1) or other species (0) in relation to environmental features at different scales (500 m and 20 km) in the Iberian Peninsula. Estimates and 95% confidence intervals were assessed. In bold, effects that received significant support (i.e. the 95% CI does not overlap zero). (*) indicates the variables that were significant in some alternative models but not in the average model. The deviance explained by the averaged model is 5.83%.

Variable	Estimate	2.5% CI	97.5% CI
Intercept	0.24	-0.11	0.58
Distance to landfills	-0.08	-0.45	0.30
Elevation500m (*)	0.36	-0.01	0.72
Precipitation500m	-0.20	-0.57	0.17
NDVI500m	-0.13	-0.51	0.25
Urban500m	-0.22	-0.58	0.13
Mosaic crops500m	-0.13	-0.57	0.32
Grasslands500m	0.29	-0.13	0.71
Wetlands500m	-0.13	-0.49	0.23
Dry crops20km	0.22	-0.16	0.60
Irrigated crops20km	0.25	-0.11	0.60
Forests20km	-0.23	-0.67	0.20
Shrublands20km	-0.23	-0.61	0.14
AC	0.18	-0.18	0.53
