# Supplementary material for: Large-scale interspecific associations and ecological context shape communal roosts of Western jackdaw (Coloeus monedula)
Source: PLoS One. 2026 May 20;21(5):e0346626. doi: 10.1371/journal.pone.0346626 (PMC13189308; doi:10.1371/journal.pone.0346626)
Supplement: S19 Table — Estimates and 95% confidence intervals were assessed. In bold, effects that received significant support (i.e., the 95% CI does not overlap zero). (PDF) [file pone.0346626.s019.pdf]

S19 Table. Alternative binomial GLM models (ΔAICc < 2) of roosting dominance by western jackdaws (*Coloeus monedula*) (1) or other species (0) in relation to environmental features at different scales (500m and 20km) in the Iberian Peninsula. Estimates and 95% confidence intervals were assessed. In bold, effects that received significant support (i.e. the 95% CI does not overlap zero).

**Variable**	**Estimate**	**2.5% CI**	**97.5% CI**
Intercept	0.24	-0.10	0.59
**Elevation500m**	0.37	0.01	0.73
Grasslands500m	0.32	-0.10	0.73
Irrigated crops20km	0.28	-0.07	0.63
Intercept	0.28	-0.06	0.62
**Elevation500m**	0.34	0.004	0.68
Urban500m	-0.25	-0.58	0.09
Intercept	0.25	-0.09	0.58
Elevation500m	0.32	-0.01	0.66
Intercept	0.23	-0.11	0.56
**Elevation500m**	0.40	0.04	0.76
Irrigated crops20km	0.24	-0.10	0.58
Intercept	0.22	-0.12	0.56
**Elevation500m**	0.35	0.01	0.69
Shrublands20km	0.26	-0.62	0.11
Intercept	0.27	-0.07	0.61
Elevation500m	0.28	-0.06	0.63
Grasslands500m	0.27	-0.14	0.67
Intercept	0.24	-0.10	0.58
Elevation500m	0.32	-0.02	0.65
Precipitation500m	-0.23	-0.58	0.13
Intercept	0.24	-0.12	0.56
Elevation500m	0.31	0.01	0.69
Grasslands500m	0.27	-0.14	0.67
Shrublands20km	-0.26	-0.62	0.11
Intercept	0.21	-0.13	0.55

**Elevation500m**	0.39	0.04	0.74
Forests20km	-0.27	-0.70	0.15
Intercept	0.21	-0.13	0.56
**Elevation500m**	0.39	0.04	0.75
Precipitation500m	-0.22	-0.59	0.14
Irrigated crops20km	0.23	-0.11	0.57
Intercept	0.23	-0.12	0.58
**Elevation500m**	0.36	0.002	0.73
Precipitation500m	-0.20	-0.57	0.17
Grasslands500m	0.30	-0.12	0.71
Irrigated crops20km	0.27	-0.07	0.62
Intercept	0.25	-0.09	0.59
**Elevation500m**	0.36	0.02	0.70
Urban500m	-0.21	-0.56	0.13
Shrublands20km	-0.22	-0.59	0.15
Intercept	0.22	-0.12	0.57
**Elevation500m**	0.38	0.01	0.74
Grasslands500m	0.31	-0.10	0.72
Irrigated crops20km	0.24	-0.12	0.60
Shrublands20km	-0.21	-0.58	0.17
Intercept	0.25	-0.09	0.60
**Elevation500m**	0.41	0.05	0.77
Urban500m	-0.21	-0.55	0.13
Irrigated crops20km	0.20	-0.15	0.55
Intercept	0.23	-0.11	0.58
**Elevation500m**	0.38	0.02	0.75
Grasslands500m	0.32	-0.10	0.73
Irrigated crops20km	0.29	-0.05	0.64
AC	0.19	-0.16	0.54
Intercept	0.24	-0.09	0.58
Grasslands500m	0.32	-0.08	0.72
Dry crops20km	0.25	-0.09	0.59
Intercept	0.23	-0.12	0.58

Elevation500m	0.35	-0.01	0.71
Grasslands500m	0.25	-0.15	0.66
Forests20km	-0.26	-0.69	0.16
Intercept	0.23	-0.10	0.57
Grasslands500m	0.32	-0.08	0.72
Intercept	0.26	-0.08	0.61
Elevation500m	0.34	-0.004	0.67
Precipitation500m	-0.19	-0.56	0.17
Urban500m	-0.22	-0.56	0.13
Intercept	0.24	-0.11	0.59
**Elevation500m**	0.40	0.04	0.75
Urban500m	-0.22	-0.56	0.13
Forests20km	-0.23	-0.66	0.20
Intercept	0.26	-0.09	0.60
Elevation500m	0.28	-0.06	0.62
Precipitation500m	-0.21	-0.57	0.15
Grasslands500m	0.25	-0.16	0.65
Intercept	0.21	-0.13	0.55
**Elevation500m**	0.41	0.05	0.77
Irrigated crops20km	0.20	-0.15	0.55
Shrublands20km	-0.21	-0.58	0.16
Intercept	0.21	-0.13	0.55
**Elevation500m**	0.42	0.06	0.77
Irrigated crops20km	0.25	-0.09	0.59
AC	0.19	-0.16	0.54
Intercept	0.22	-0.13	0.56
**Elevation500m**	0.34	0.001	0.68
Precipitation500m	-0.19	-0.55	0.17
Shrublands20km	-0.22	-0.59	0.15
Intercept	0.27	-0.07	0.61
**Elevation500m**	0.35	0.01	0.69
Urban500m	-0.25	-0.59	0.09
AC	0.18	-0.17	0.52

Intercept	0.20	-0.15	0.54
**Elevation500m**	0.40	0.05	0.75
Forests20km	-0.23	-0.65	0.20
Shrublands20km	-0.22	-0.59	0.15
Intercept	0.22	-0.12	0.56
Grasslands500m	0.38	-0.03	0.78
Dry crops20km	0.29	-0.06	0.64
Irrigated crops20km	0.22	-0.10	0.55
Intercept	0.29	-0.06	0.63
Elevation500m	0.31	-0.04	0.66
Urban500m	-0.20	-0.55	0.15
Grasslands500m	0.20	-0.22	0.62
Intercept	0.24	-0.10	0.58
Elevation500m	0.33	-0.01	0.67
AC	0.17	-0.18	0.51
Intercept	0.22	-0.13	0.57
**Elevation500m**	0.40	0.03	0.77
Grasslands500m	0.30	-0.11	0.71
Irrigated crops20km	0.24	-0.12	0.60
Forests20km	-0.18	-0.62	0.26
Intercept	0.24	-0.11	0.58
Elevation500m	0.29	-0.12	0.70
Grasslands500m	0.33	-0.08	0.75
Dry crops20km	0.16	-0.23	0.55
Irrigated crops20km	0.28	-0.06	0.63
Intercept	0.26	-0.09	0.61
**Elevation500m**	0.38	0.02	0.74
Urban500m	-0.14	-0.50	0.22
Grasslands500m	0.27	-0.16	0.70
Irrigated crops20km	0.25	-0.11	0.60
Intercept	0.20	-0.14	0.54
**Elevation500m**	0.44	0.07	0.80
Irrigated crops20km	0.19	-0.16	0.55

Forests20km	-0.21	-0.64	0.23
Intercept	0.21	-0.14	0.55
**Elevation500m**	0.37	0.02	0.73
Precipitation500m	-0.19	-0.56	0.18
Forests20km	-0.23	-0.66	0.21
Intercept	0.21	-0.13	0.55
Grasslands500m	0.33	-0.07	0.72
Shrublands20km	-0.23	-0.60	0.13
Intercept	0.22	-0.11	0.56
Precipitation500m	-0.21	-0.57	0.15
Grasslands500m	0.30	-0.10	0.70
Intercept	0.21	-0.11	0.55
Dry crops20km	0.25	-0.09	0.59
Intercept	0.27	-0.07	0.61
**Elevation500m**	0.36	0.02	0.70
NDVI500m	-0.14	-0.52	0.23
Urban500m	-0.28	-0.64	0.07
Intercept	0.26	-0.08	0.60
Elevation500m	0.29	-0.05	0.64
Grasslands500m	0.26	-0.14	0.67
AC	0.17	-0.18	0.51
Intercept	0.21	-0.14	0.56
Elevation500m	0.36	-0.002	0.72
Grasslands500m	0.26	-0.14	0.66
Shrublands20km	-0.23	-0.60	0.14
Forests20km	-0.21	-0.64	0.21
Intercept	0.23	-0.11	0.58
**Elevation500m**	0.38	0.01	0.74
NDVI500m	-0.11	-0.49	0.26
Grasslands500m	0.34	-0.08	0.77
Irrigated crops20km	0.28	-0.07	0.63
Intercept	0.28	-0.06	0.62
Elevation500m	0.32	-0.03	0.66

Urban500m	-0.28	-0.63	0.07
Wetlands500m	-0.13	-0.49	0.23
Intercept	0.23	-0.11	0.57
Precipitation500m	-0.21	-0.58	0.16
Grasslands500m	0.30	-0.10	0.70
Dry crops20km	0.24	-0.10	0.59
Intercept	0.23	-0.11	0.58
Elevation500m	0.30	-0.04	0.65
Precipitation500m	-0.17	-0.54	0.19
Grasslands500m	0.26	-0.15	0.66
Shrublands20km	-0.23	-0.61	0.14
Intercept	0.23	-0.12	0.58
**Elevation500m**	0.36	0.001	0.73
Mosaic crops500m	-0.11	-0.55	0.33
Grasslands500m	0.32	-0.09	0.74
Irrigated crops20km	0.28	-0.07	0.63
Intercept	0.24	-0.09	0.58
Elevation500m	0.26	-0.11	0.64
Dry crops20km	0.12	-0.27	0.51
Intercept	0.26	-0.09	0.60
Elevation500m	0.34	-0.003	0.67
Urban500m	-0.26	-0.60	0.08
Mosaic crops500m	-0.14	-0.58	0.30
Intercept	0.20	-0.14	0.55
**Elevation500m**	0.40	0.04	0.75
Forests20km	-0.27	-0.69	0.16
AC	0.16	-0.19	0.51
Intercept	0.24	-0.10	0.59
**Elevation500m**	0.42	0.06	0.78
Urban500m	-0.21	-0.56	0.13
Irrigated crops20km	0.22	-0.13	0.56
AC	0.19	-0.16	0.54
Intercept	0.24	-0.11	0.59

**Elevation500m**	0.40	0.04	0.76
Precipitation500m	-0.20	-0.57	0.18
Urban500m	-0.18	-0.53	0.17
Irrigated crops20km	0.20	-0.14	0.55
Intercept	0.26	-0.09	0.60
Elevation500m	0.33	-0.02	0.67
Urban500m	-0.16	-0.51	0.19
Grasslands500m	0.22	-0.20	0.64
Shrublands20km	-0.24	-0.61	0.14
Intercept	0.24	-0.10	0.59
Distance to landfills	-0.08	-0.45	0.29
**Elevation500m**	0.39	0.02	0.76
Grasslands500m	0.33	-0.09	0.74
Irrigated crops20km	0.27	-0.07	0.62
