# Supplementary figures and images for: Large-scale interspecific associations and ecological context shape communal roosts of Western jackdaw (Coloeus monedula)
Source: PLoS One. 2026 May 20;21(5):e0346626. doi: 10.1371/journal.pone.0346626 (PMC13189308; doi:10.1371/journal.pone.0346626)

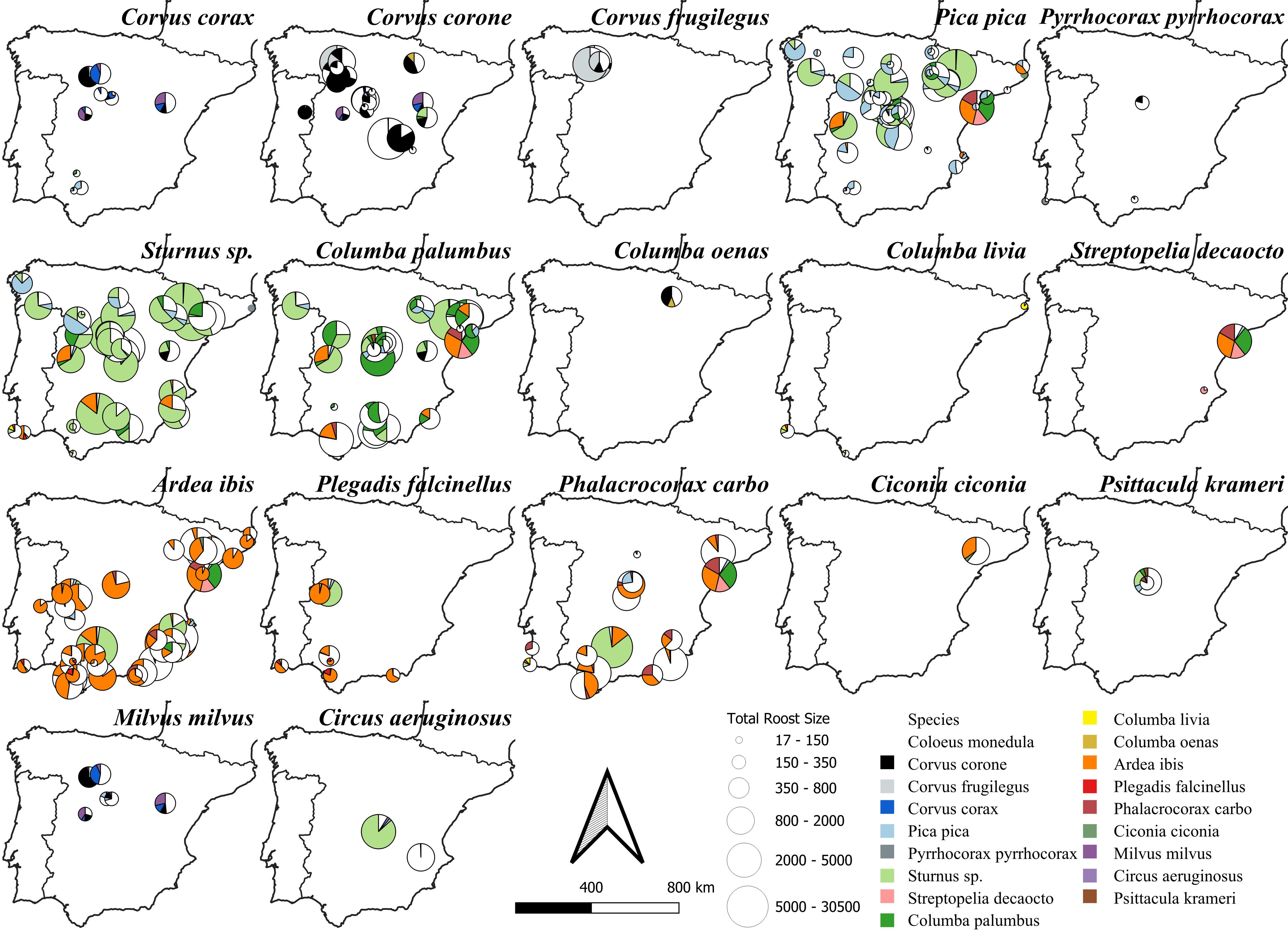

Supplement: S1 Fig — Point size is proportional of the overall roost size of the abundance of all roost abundance, and the colour corresponded to the different species within the jackdaw roosts. Country borders were obtained from Natural Earth (https://www.naturalearthdata.com/). (TIFF) [file pone.0346626.s020.tiff]

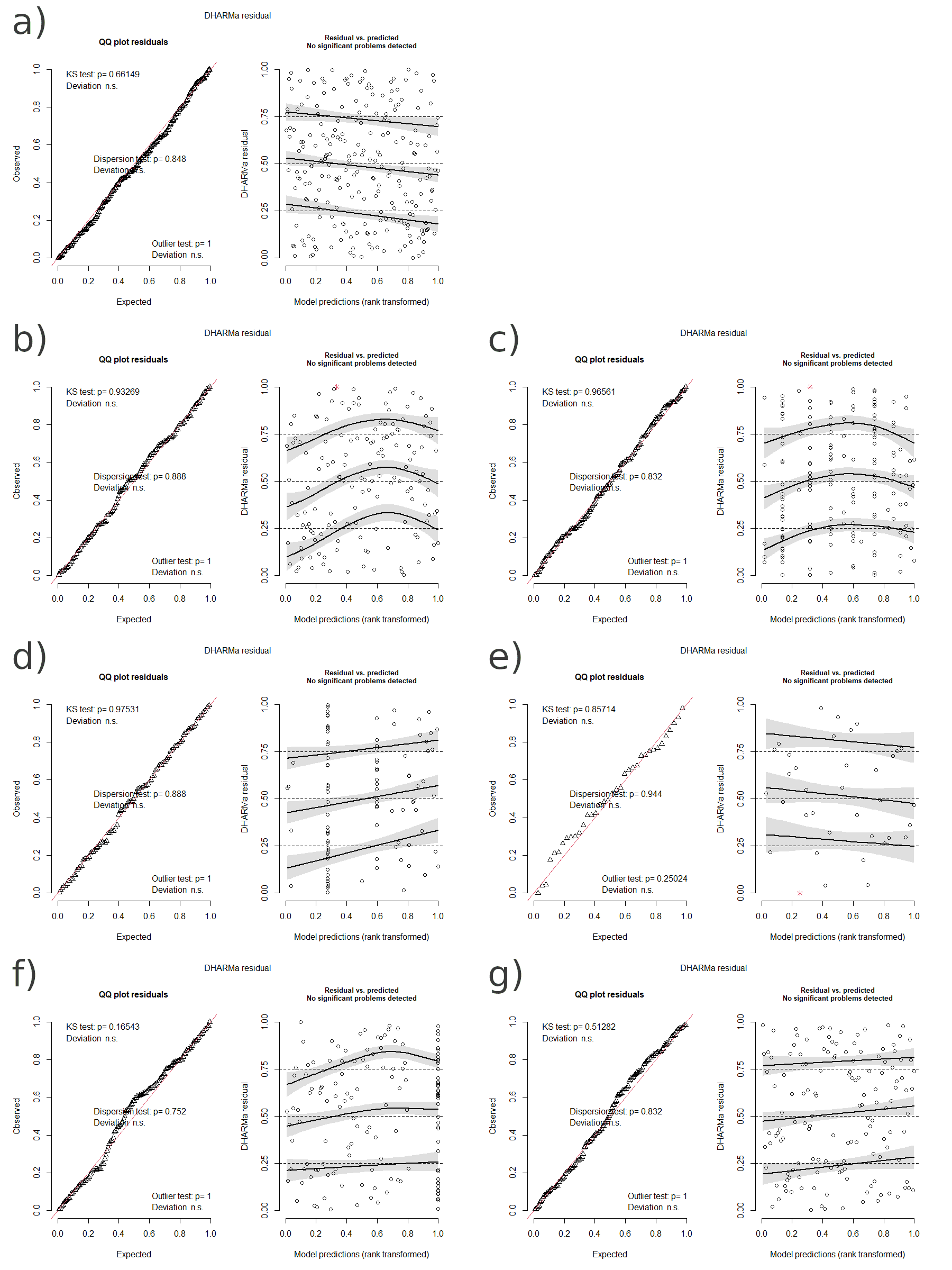

Supplement: S2 Fig — (TIFF) [file pone.0346626.s021.tiff]
